# The anti-diabetic PPARγ agonist Pioglitazone inhibits cell proliferation and induces metabolic reprogramming in prostate cancer

**DOI:** 10.1186/s12943-025-02320-y

**Published:** 2025-05-05

**Authors:** Emine Atas, Kerstin Berchtold, Michaela Schlederer, Sophie Prodinger, Felix Sternberg, Perla Pucci, Christopher Steel, Jamie D. Matthews, Emily R. James, Cécile Philippe, Karolína Trachtová, Ali A. Moazzami, Nastasiia Artamonova, Felix Melchior, Torben Redmer, Gerald Timelthaler, Elena E. Pohl, Suzanne D. Turner, Isabel Heidegger, Marcus Krueger, Ulrike Resch, Lukas Kenner

**Affiliations:** 1 Department of Pathology, Medical University of Vienna, Vienna, Austria; 2 Christian Doppler Laboratory for Applied Metabolomics (CDL-AM), Medical University of Vienna, Vienna, Austria; 3 Department of Biomedical Imaging and Image-Guided Therapy, Division of Nuclear Medicine, Medical University of Vienna, Vienna, Austria; 4 University of Vienna, Vienna, Austria; 5 Department of Biological Sciences and Pathobiology, Unit of Physiology and Biophysics, University of Veterinary Medicine, Vienna, Austria; 6 Department of Nutritional Science, University of Vienna, Vienna, Austria; 7 Division of Cellular and Molecular Pathology, Department of Pathology, University of Cambridge, Cambridge, UK; 8 Central European Institute of Technology, Masaryk University, Brno, 62500 Czech Republic; 9 Department of Molecular Sciences, Swedish University of Agricultural Sciences, 75007 Uppsala, Sweden; 10 Department of Urology, Medical University of Innsbruck, Innsbruck, Austria; 11 Unit of Laboratory Animal Pathology, Institute of Pathology, University of Veterinary Medicine Vienna, Vienna, Austria; 12 Center for Cancer Research, Medical University of Vienna, Vienna, Austria; 13 Faculty of Medicine, Masaryk University, Brno, Czech Republic; 14 Institute for Genetics, Cologne Excellence Cluster of Cellular Stress Responses in Aging-Associated Diseases (CECAD), Cologne, Germany; 15 Center of Physiology and Pharmacology, Department of Vascular Biology and Thrombosis Research, Medical University of Vienna, Vienna, Austria; 16 Center for Biomarker Research in Medicine GmbH (CBmed), Graz, Austria; 17 Department of Molecular Biology, Umeå University, Umea, Sweden

**Keywords:** Metabolic rewiring, Type 2 diabetes mellitus (T2DM), PPAR agonists, Cancer therapy, Oxygen consumption rate, Extracellular acidification, Energy metabolism

## Abstract

Prostate cancer (PCa) and Type 2 diabetes (T2D) often co-occur, yet their relationship remains elusive. While some studies suggest that T2D lowers PCa risk, others report conflicting data. This study investigates the effects of peroxisome proliferator-activated receptor (PPAR) agonists Bezafibrate, Tesaglitazar, and Pioglitazone on PCa tumorigenesis. Analysis of patient datasets revealed that high *PPARG* expression correlates with advanced PCa and poor survival. The PPARγ agonists Pioglitazone and Tesaglitazar notably reduced cell proliferation and PPARγ protein levels in primary and metastatic PCa-derived cells. Proteomic analysis identified intrinsic differences in mTORC1 and mitochondrial fatty acid oxidation (FAO) pathways between primary and metastatic PCa cells, which were further disrupted by Tesaglitazar and Pioglitazone. Moreover, metabolomics, Seahorse Assay-based metabolic profiling, and radiotracer uptake assays revealed that Pioglitazone shifted primary PCa cells' metabolism towards glycolysis and increased FAO in metastatic cells, reducing mitochondrial ATP production. Furthermore, Pioglitazone suppressed cell migration in primary and metastatic PCa cells and induced the epithelial marker E-Cadherin in primary PCa cells. In vivo*,* Pioglitazone reduced tumor growth in a metastatic PC3 xenograft model, increased phosho AMPKα and decreased phospho mTOR levels. In addition, diabetic PCa patients treated with PPAR agonists post-radical prostatectomy implied no biochemical recurrence over five to ten years compared to non-diabetic PCa patients. Our findings suggest that Pioglitazone reduces PCa cell proliferation and induces metabolic and epithelial changes, highlighting the potential of repurposing metabolic drugs for PCa therapy.

**Graphical Abstract:**

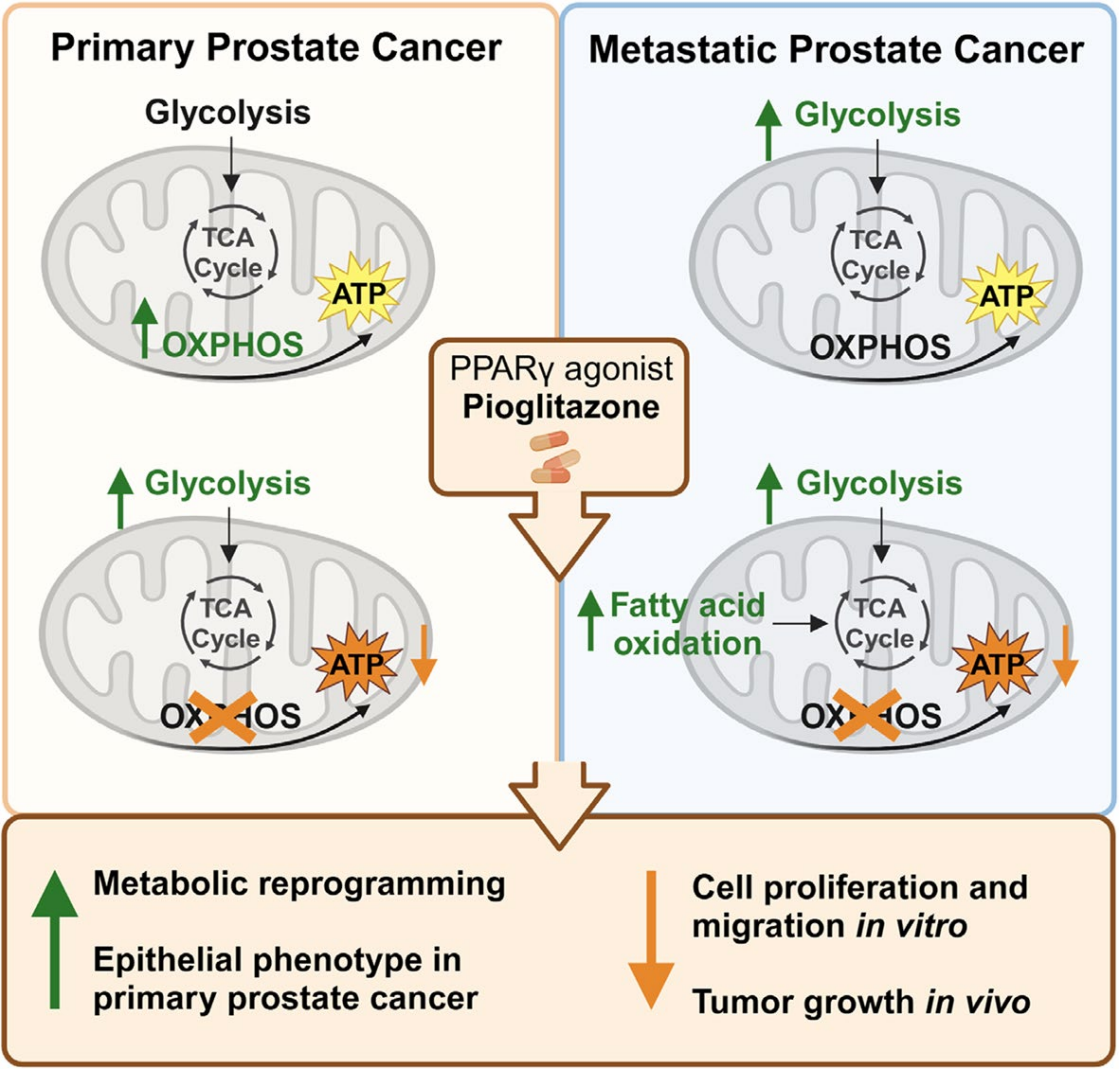

**Supplementary Information:**

The online version contains supplementary material available at 10.1186/s12943-025-02320-y.

## Introduction

Prostate cancer (PCa) is the second most prevalent cancer in men, accounting for approximately 29% of cancer incidence in Western countries [1]. The function of the normal prostate gland and the pathogenesis of PCa are primarily driven by androgens and their interaction with the androgen receptor (AR) [2, 3]. Testosterone and 5α-dihydrotestosterone (DHT), the primary androgens, bind to the AR, leading to its dimerization, nuclear translocation, and subsequent initiation of target gene transcription in collaboration with co-activators [3]. Elevated androgen levels can contribute to developing prostatic intraepithelial neoplasia (PIN), a precursor to primary PCa [4]. In the past years, the therapeutic landscape of metastatic PCa has changed significantly, moving toward androgen deprivation therapy (ADT) (Abiraterone, Enzalutamide, Apalutamide) or ADT in combination with Docetaxel chemotherapy. Nevertheless, almost all patients relapse after 2–3 years of entering a so-called castration-resistant stage (CRPCa), which highlights the urgent need for novel treatment strategies [2, 4–6]. Besides being at risk of developing PCa, men are also more prone to type 2 diabetes (T2D) than women [1, 7]. T2D is the most common metabolic disorder diagnosed worldwide. It is a heterogeneous, chronic disease characterized by increased blood glucose levels (hyperglycemia) and deficient insulin secretion by pancreatic ß-cells, often due to insulin resistance [7–11]. The concurrent prevalence of PCa and T2D increases with age and obesity, presenting significant epidemiologic and therapeutic challenges [1, 7]. The mechanistic link between PCa and T2D remains elusive since existing studies provide conflicting evidence. For instance, ADT frequently results in side effects such as insulin resistance and hyperglycemia, potentially aggravating the risk of diabetes [12]. Moreover, patients with untreated T2D or those treated with insulin or anti-hyperglycemics show higher PCa mortality compared to men without T2D [13, 14]. In contrast, Peila et al., 2020 demonstrated an inverse correlation between PCa and T2D, where men with diabetes and elevated hemoglobin A1c (HbA1c) levels—a marker for glycemic control—exhibited a lower risk of developing PCa [15]. This protective effect was also observed in men with long-term diabetes diagnosed with localized, low-grade PCa [16]. Following these latter data, the anti-diabetic drug Metformin suppressed tumor growth in preclinical studies, such as a xenograft model of androgen-dependent PCa cells [17]. Additionally, a randomized controlled trial found that thiazolidinediones (TZD), a class of T2D medications, were associated with a reduced incidence of PCa, suggesting the potential for these drugs in PCa prevention [18]. In addition to Metformin, which is commonly prescribed as a primary treatment, TZDs are employed either as standalone or supplementary therapies for T2D [19, 20]. TZDs are synthetic insulin sensitizers that act as agonists to the nuclear receptor peroxisome proliferator-activated receptor gamma (PPARγ). PPAR nuclear receptors are transcription factors that function as nutrient sensors of fatty acid metabolites to regulate lipid- and glucose metabolism in a cell-type and organ-specific manner [19, 20]. PPAR alpha (PPARα) is predominantly expressed in tissues with a high capacity for fatty acid oxidation (FAO), such as liver, heart, or brown adipose tissue [21]. Bezafibrate, a pharmacological PPARα agonist with anti-hyperlipidemic properties, is used in the management of hypertriglyceridemia and cardiovascular disease [22–25]. PPARγ is more frequently found in adipose tissue or spleen, where it plays a significant role in adipogenesis and energy balance [21]. The dual PPARα/γ agonist Tesaglitazar has also shown superior efficacy in enhancing glucose and lipid metabolism than selective PPARγ agonists [26, 27]. Furthermore, the PPARγ agonist Pioglitazone increases glucose uptake, reduces free fatty acids in plasma, enhances insulin signalling, and has a triglyceride-reducing effect [28].

This study aimed to decipher common and specific molecular pathways targeted by the PPAR agonists Bezafibrate, Tesaglitazar, and Pioglitazone in primary and metastatic PCa cells. We show that high *PPARG* mRNA expression correlates with inferior survival for PCa patients. Notably, the PPARγ agonist Pioglitazone reduced PCa cell proliferation in vitro and in vivo, induced metabolic reprogramming, and promoted an epithelial phenotype in PCa. Our findings suggest that using metabolic drugs such as PPARγ agonists could improve PCa treatment outcomes by reducing tumor growth, reprogramming metabolic pathways, and promoting a more benign epithelial phenotype.

## Materials and Methods

All the materials and consumables of this study are listed in Tab .1.

**Table 1:** Materials and consumables

REAGENT or RESOURCE	SOURCE	IDENTIFIER
**Antibodies for Western blot**		
Mouse monoclonal β-Actin (8H10D10)	Cell Signaling	#3700
Rabbit monoclonal AMPKα (D5 A2)	Cell Signaling	#5831
Rabbit monoclonal AR [EPR1535(2)]	Abcam	#ab133273
Rabbit monoclonal E-Cadherin (24E10)	Cell Signaling	#3195
Rabbit monoclonal phospho AMPKα (Thr172) (40H9)	Cell Signaling	#2535
Rabbit monoclonal PPARγ (C26H12)	Cell Signaling	#2435
Rabbit polyclonal mTOR	Cell Signaling	#2972
Rabbit polyclonal phospho mTOR (Ser2448)	Cell Signaling	#2971
**Antibodies for immunofluorescence (IF)**		
Anti-rabbit—Alexa fluor 488	Invitrogen	#A11008
Rabbit monoclonal E-Cadherin (24E10)	Cell Signaling	#3195
**Antibodies for immunohistochemistry (IHC)**		
Rabbit monoclonal Ki67 (D3B5)	Cell Signaling	#12202
Rabbit polyclonal CC3 (Asp175)	Cell Signaling	#9661
Rabbit monoclonal phospho AMPKα (Thr 172)	Cell Signaling	#2535
Rabbit monoclonal phospho mTOR (Ser2448)	Cell Signaling	#2976
**Chemicals, peptides, and recombinant proteins**		
2-deoxy-glucose (2-DG)	Sigma Aldrich	#D8375
Annexin V	Thermo Fisher Scientific	#R37174
Antimycin A	Sigma Aldrich	#A8674
Bezafibrate	Sigma Aldrich	#B7273
Bodipy 493/503	Invitrogen	#D392
Bovine Serum Albumin (BSA)	Sigma Aldrich	#A9418
Clarity Western ECL Substrate	Biorad	#170–561
DAPI	Sigma Aldrich	#D9542
Fat-free BSA	Carl Roth	#00.52
FCCP	Sigma Aldrich	#C2920
Fetal calf serum (FCS)	Gibco	#10500064
Fluorescence mounting media	DAKO	#S3023
Formaldehyde	Sigma Aldrich	#252549
Gibco DMEM, no glucose	Thermo Fisher Scientific	#11966025
Glucose	Agilent	#103577–100
Glutamine	Agilent	#103579–100
Laemmli buffer	Biorad	#1610747
Nitrocellulose membrane	Amersham Protran	#10600001
Oligomycin	Sigma Aldrich	#495455
PBS	Carl Roth	#1105
Penicillin/streptomycin	Gibco	#15140122
Pioglitazone	Sigma Aldrich	#CDS021593
Poly-L-lysine	Sigma Aldrich	#P2636
PonceauS	Merck	#15,927
Protease inhibitor cOmplete Mini	Roche	#11836153001
Universal block	DCS Innovative Diagnostik Systeme	#UL123R100
Pyruvate	Agilent	#103578–100
Resazurin	Sigma Aldrich	#B70717
RIPA buffer	Boston BioProducts	#B- 115
Rotenone	Sigma Aldrich	#557368
RPMI	Gibco	#52400–025
Seahorse XF RPMI medium	Agilent	#103576–100
Tesaglitazar	Sigma Aldrich	#SML1369
Tween20	Sigma Aldrich	#P1379
**Commercial assays**		
Coomassie Blue Bradford assay	Thermo Fisher Scientific	#23236
CyQUANT NF cell proliferation assay kit	Thermo Fisher Scientific	#C35006
cDNA synthesis kit	NEB	#M0368
Luna qRT-PCR master mix	NEB	#M3003
RNA isolation kit	NEB	#TS2010S
Punch kit	Merck	#MAMP09608
**Experimental models: Cell lines**		
Human: 22RV1 cells	ATCC	CRL- 2505
Human: PC3 cells	ATCC	CRL- 1435
**Experimental models: mice**		
NSG mice	Charles River	
**Oligonucleotides**		
*AR*: 5’− 3’ forward AGAGTGCCCTATCCCAGTCC	This paper	N/A
*AR*: 5’− 3’ reverse TGGCAGTCTCCAAACGCAT	This paper	N/A
*AR*: 5’− 3’ forward GTCTTCCCCTCCATCGTG	This paper	N/A
*AR*: 5’− 3’ reverse AGGGTGAGGATGCCTCTCTT	This paper	N/A
*PPARA*: 5’− 3’ forward GCTGTCACCACAGTAGCTTG	This paper	N/A
*PPARA*: 5’− 3’ reverse AGCTTCCAGAACTATCCTCGC	This paper	N/A
*PPARD*: 5’− 3’ forward CAGAGCTATGACTGGGCCTG	This paper	N/A
*PPARD*: 5’− 3’ reverse CTCCGGGAGAGGTCTGTGTA	This paper	N/A
*PPARG*: 5’− 3’ forward GTGCAATCAAAGTGGAGCCTG	This paper	N/A
*PPARG*: 5’− 3’ reverse TCCGGAAGAAACCCTTGCATC	This paper	N/A
**Software and algorithms**		
Enrichr	Chen, EY. Et al., 2013 [29] Kuleshov, MV. et al., 2016 [30] Xie, Z. et al., 2021 [31]	https://maayanlab.cloud/Enrichr/
FlowJo _V10	FlowJo BD Biosciences	https://www.flowjo.com/solutions/flowjo
Gen5™ Data Analysis Software	Biotek	N/A
GraphPad Prism 8	GraphPad by Dotmatics	https://www.graphpad.com/
ImageLab	Biorad	https://www.bio-rad.com/de-at/product/image-lab-software?ID=KRE6P5E8Z
IncuCyte	Sartorius	https://www.sartorius.com/en/products/live-cell-imaging-analysis/live-cell-analysis-software
Interacti Venn	Heberle, H. et al., 2015 [32]	https://www.interactivenn.net/
KM Plotter	Gyorffy, B. et al. 2023 [33]	https://kmplot.com/
Prostate Cancer Atlas	Bolis, M. et al. 2021 [34]	https://prostatecanceratlas.org/
ProteoWizard	Chambers, M. et al. 2012 [35]	https://proteowizard.sourceforge.io/
QuPath- 0.5.1	Bankhead et al. 2017 [36]	https://qupath.github.io/
Seahorse Wave Desktop	Agilent	https://www.agilent.com/en/product/cell-analysis/real-time-cell-metabolic-analysis/xf-software/seahorse-wave-desktop-software-740897
SR Plot	SRPLOT	https://www.bioinformatics.com.cn/en
Zen2 blue	Zeiss	https://www.micro-shop.zeiss.com/de/de/softwarefinder/software-categories/zen-blue
R packages “Survival”, “Survminer”, R-script “ggsurvplot	The Comprehensive R Archive Network	https://cran.r-project.org/web/packages
fgsea R package (version 1.22.0)	Korotkevich, G. et al., 2016 [37]	https://bioconductor.org/packages/release/bioc/html/fgsea.html
R Studio 4.2.0	Posit	https://posit.co/download/rstudio-desktop/
Perseus 1.6.15.0	MaxQuant	https://maxquant.net/perseus/
NMR Suite 10.0 profiler	ChenomX Inc, Edmonton, Canada	https://www.chenomx.com/
InstantClue 0.12.1	Nolte, H. et al., 2018 [38]	http://www.instantclue.uni-koeln.de/
msigdbr R package (version 7.5.1)	Liberrzon, A. et al., 2015 [39] Subramanian, A. et al., 2005 [40]	https://www.gsea-msigdb.org/gsea/msigdb/
DIA-NN 1.8.1	Demichev, V. et al. 2020 [41]	https://github.com/vdemichev/DiaNN/releases
Bruker Topspin 1.3	Bruker	https://www.bruker.com/en/products-and-solutions/mr/nmr-software/topspin.html
SIMCA-P + 17.0	Umetrics, Umeå, Sweden	https://www.sartorius.com/en/products/process-analytical-technology/data-analytics-software/mvda-software/simca
Other		
96-well filter plates	Merck Millipore	#MADVN6550
Axio Imager M2	ZEISS	https://www.zeiss.com
BD FACS Canto 2	BD Biosciences	https://www.bdbiosciences.com/en-us
Bruker spectrometer 600 MHz	Bruker	https://www.bruker.com
ChemiDoc MP Imaging system	BioRad	https://www.bio-rad.com
Easy nLC1000-QExactive Plus	Thermo Fisher Scientific	https://www.thermofisher.com/at/en/home.html
Gamma Counter Wizard	PerkinElmer	https://www.perkinelmer.com/de/
Imagelock 96well plates	Sartorius	#BA- 04856
Incucyte S3 live cell imaging	Sartorius	https://www.sartorius.com/
Seahorse XF Pro M cell culture Microplate	Agilent	#103774–100
Seahorse XF- 96 analyzer	Agilent	https://www.agilent.com
Superfrost microscopy slides	Epredia	#J1800 AMNZ
Synergy H1 microplate reader	Biotek	N/A

### Cell culture

The human primary PCa 22RV1 and bone marrow metastasis PC3 cells were cultured in RPMI supplemented with 10% fetal calf serum (FCS) and 1% penicillin/streptomycin (Pen/Strep) (RPMI full media) at 37 °C with 5% CO_2_.

### Prostate Cancer Atlas

The Prostate Cancer Atlas (https://prostatecanceratlas.org/) was used to assess *PPARA*, *PPARD*, and *PPARG *mRNA expression throughout different PCa stages (healthy, primary PCa, ARPC) [34]. One-way ANOVA was used to evaluate statistical significance (ns = not significant *p* > 0.05, * *p* ≤ 0.05, ** *p* ≤ 0.01, *** *p* ≤ 0.001, **** *p* ≤ 0.0001) using GraphPad Prism v8. Individual values with mean ± SD were visualized in scattered dot plots.

### Kaplan–Meier survival analyses of public datasets

To assess whether *PPARA*, *PPARD*, *PPARG*, *PTEN*, or *STAT3* expression levels affected survival probabilities of human PCa patients, we applied the Kaplan–Meier (KM) plotter tool (https://kmplot.com/analysis/), which computed probabilities of BCR-free survival based on the TCGA-PRAD study comprising survival data of PCa patients (*n* = 333) [42]. Output data were used for re-plotting of survival curves and performing Cox-regression analyses with R packages “Survival” and “Survminer” and R-script “ggsurvplot”. Combined KM plotter output was used to calculate subgroups stratified by expression levels of both genes, such as *PPARG* and *PTEN* or *PPARG* and *STAT3*. Groups were automatically separated, and the calculated, best-performing, and most significant threshold was used as a cut-off. Hazard ratios and *p*-values were retrieved from Cox-regression analyses.

### Cell viability and proliferation assay

Cell viability and – proliferation were measured via the resazurin assay. 3 to 5 * 10^3^ cells/well were seeded into a 96-well plate. After cell attachment, cells were treated with various concentrations of the PPAR agonists Bezafibrate, Tesaglitazar, Pioglitazone (10, 50, 100, 200, 300 µM) or vehicle control (0.2% DMSO). Cell viability was determined after 72 h, whereas cell proliferation was measured between 24 and 96 h. For both experiments, resazurin diluted 1:5 in RPMI full media was added to the cells and incubated for 2 h at 37 °C and 5% CO_2_. Fluorescence with 530/570 nm excitation filters and emission at 580/620 nm was measured in the Synergy H1 microplate reader (Biotek) and analyzed in the Gen5 data analysis software. One-way ANOVA was used to evaluate statistical significance (ns = not significant *p* > 0.05, * *p* ≤ 0.05, ** *p* ≤ 0.01, *** *p* ≤ 0.001, **** *p* ≤ 0.0001). The graphs were plotted in GraphPad Prism v8.

### CyQUANT NF cell proliferation assay

For more sensitive cell proliferation measurements, 5 * 10^3^ cells were seeded into a poly-L-lysine coated 96-well plate and treated with 100 µM Bezafibrate, Tesaglitazar, Pioglitazone, and vehicle control (0.2% DMSO) for 72 h in RPMI full media under standard culture conditions. 0.2% of CyQuant NF dye reagent was prepared in 1 × HBSS buffer, added to the wells in a 1:1 ratio, and incubated at 37 °C for 60 min. The fluorescence signal was measured in the Synergy H1 microplate reader (Biotek) at 485/530 nm excitation/emission wavelength. One-way ANOVA was used to evaluate statistical significance (ns = not significant *p* > 0.05, * *p* ≤ 0.05, ** *p* ≤ 0.01, *** *p* ≤ 0.001, **** *p* ≤ 0.0001). The graphs were plotted in GraphPad Prism v8.

### RNA isolation and quantitative reverse transcription PCR (qRT-PCR)

Total RNA was isolated from cell pellets according to the manufacturer's (NEB) protocol. cDNA was reverse transcribed from 1 µg of RNA, and 1:10 dilutions of the cDNA were used in qRT-PCR reactions containing 0.25 µM of forward and reversed primers and Luna qRT-PCR master mix. For quantitative analysis, β-Actin was used as a reference gene. Gene expression was calculated using the delta-delta Ct method, as described previously [43].

### Protein isolation and Western blot

Cell protein extracts were prepared from cell pellets resuspended and lysed in 1 × RIPA Buffer. After 15 min of incubation at 4 °C, the samples were sonicated for 5 cycles/30 s to disrupt DNA and then centrifuged at full speed at 4 °C for 15 min. The protein concentration was determined using the Coomassie Blue Bradford assay with BSA as a standard. The sample concentration was adjusted to 1 µg/µl with lysis buffer, reduced, and denatured in 1 × Laemmli buffer by heating at 95 °C for 10 min. Proteins were separated on custom-made SDS-PAGE (10%) and blotted onto nitrocellulose membranes. The membranes were blocked with 5% BSA in 1 × TBST + 0.02% NaN_3_ for 1 h at room temperature (RT) and incubated overnight at 4 °C with primary antibody. After washing and incubation with HRP-conjugated secondary antibodies for 1 h, the membranes were developed using Clarity Western ECL Substrate, imaged, processed, and quantified with the ChemiDoc MP Imaging system (Biorad) and ImageLab software.

### Annexin V staining

To assess apoptosis throughout 24 to 72 h, 3 * 10^5^ of 22RV1 and 1.5 * 10^5^ of PC3 cells were seeded and treated with 100 µM of each PPAR agonist or the vehicle control (0.2% DMSO) in RPMI full media. For each time point, the cells were harvested by trypsinization, washed with 1 × PBS, and stained with Annexin V for 15 min according to product instructions. Thereafter, the cells were stained with 1 µg/ml DAPI. Annexin V-positive cells in the FITC channel were gated against the DAPI-positive cells in the Pacific Blue channel. The FACS data were acquired with the BD FACS Canto 2 (BD Biosciences) and analyzed via the FlowJo _V10 software.

### Sample preparation for LC–MS/MS analysis

Cells were incubated for 24 h in full media with 100 µM Bezafibrate, Tesaglitazar, Pioglitazone, or vehicle control (0.2% DMSO). The treated cells were washed twice with 1 × PBS, shock-frozen at − 80°C, scraped from plates after the addition of 2 × Lämmli buffer (100 µl/well), denatured for 10 min at 95 °C, sonicated, heated again and insoluble material was removed after centrifugation for 10 min at 13,000 g. Protein content was estimated based on Coomassie-stained SDS-PAGE and 10 µg in replica was transferred to strip-PCR-tubes, reduced (10 mM TCEP, tris(2-carboxyethyl) phosphine) Thermo Fisher Scientific) and carbamidomethylated (5 mM, CAA, 2-chloroacetamide), Merck) by incubation for 10 min at 70 °C, and processed for on-bead digestion using the SP3 method with Lys-C and trypsin at 1:100 enzyme-to-substrate ratios in 50 mM ammonium bicarbonate for 6 h at 37 °C [44]. Digests were acidified (1% formic acid, FA), desalted, and concentrated with SDB-RPS stage tips with 2 layers of styrene–divinylbenzene resin (AttractSPE Disk, Affinisep). Peptides were eluted from the SDB-RPS membrane (1% ammonia in 60% acetonitrile (ACN), 15 min at RT) in a 96-well plate, dried in a speed vac, and reconstituted in 13 µl resuspension buffer (2% ACN, 5% FA). LC–MS/MS analysis was performed on an Easy nLC1000-QExactive Plus setting. 2 µl (corresponding to approximately 2 µg) peptides were separated on an in-house made 20 cm analytical column (75 µm diameter, Poroshell 120 (2.7 µm) C18 resin), column temperature 50 °C by a 90 min gradient (250 nl/min flow rate) in a binary solvent system (A: 0.1% FA in water and B: 80% ACN in stepwise gradient to 25% B in 68 min, 12 min to 70% B and finally 8 min to 95% B). The mass spectrometer was operated in a data-independent mode using a staggered (overlapping) window pattern to acquire 25 × 24 m/z (400–1000 m/z) precursor isolation window DIA spectra (17.500 resolution, AGC target 1e6, maximum injection time 60 ms, 27 NCE, fixed first mass 200 m/z). Full-MS precursor spectra (target range ± 15 m/z, at resolution 35.000, AGC target 1e6, injection time 60 ms, scan range 385–1015 m/z) were interspersed every 25 MS/MS spectra. DIA-Raw files were demultiplexed using ProteoWizard with “Apply peak picking”, “Demultiplex overlapping spectra”, and “Optimization” enabled, and “Intensity encoding precision” set to 32-bit and mzML were analyzed with DIA-NN version 1.8.1 using the canonical Uniprot human fasta database (download 2022, 20,146 entries) and library free (predicted) search [41, 45]. Missed cleavage and maximum variable modifications (methionine oxidation, N-terminal acetylation, and cysteine-carbamidomethylation) were set to 1, peptide length was set to 7–30 amino acids, precursor charge range 1–4, and m/z range form 300–1800 m/z. Protein inference was on proteins (from Fasta), neural network classifier set to “double-pass-mode”, quantification strategy “robust LD (high acquisition)”, “use isotopologues” and “MBR” enabled. Max.LFQ abundance for protein groups was calculated from the “precursor.translated” column of the DIANN.tsv output with the DIANN R package for MS2-centric methods, filtered for global q-value (Lib.PG.Q.value < 0.01 and “count.stripped.sequence” > 1. MaxLFQ columns were subsequently analyzed with Perseus (v 1.6.15.0) and visualized in Instant clue (v 0121) [38, 46, 47].

### Statistical data analysis of LC–MS/MS analysis

Initially, protein identifications for each condition (22RV1 or PC3 vehicle control or PPAR agonist treated, Suppl. Tab. 1) were compared in qualitative Venn diagrams created with InteractiVenn (www.interactivenn.net) [32]. At first, we compared the basal proteome repertoire of 22RV1 and PC3 after filtering for at least 2 valid values (Suppl. Tab. 1). Based on this filtering, we used the Enrich tool and the MSig database to perform enrichment analysis of commonly detected (3294) and cell lines-specific proteins (312 22RV1 only, 2147 PC3 only) (Suppl. Tab. 2). The enrichment ratio was calculated based on the number of detected proteins and the total number of proteins within the respective pathway. 3294 common proteins eligible for comparative analysis were then subjected to unpaired Welch T-tests with permutation-based FDR correction (*q* < 0.05) enabled. All 917 significantly changed proteins (-log_10_ *p* ≥ 1.3 and *q* < 0.05) are reported in Suppl. Tab. 1 S1_03 and proteins meeting the fold change cut-off criteria of 1 are summarized in Suppl. Tab. 1 S1_04. Volcano plots were generated in Instant Clue with proteins meeting the fold change cut-off criteria of 1 color-coded. The log_2_ fold change and *p*-value of differentially expressed proteins in 22RV1 and PC3 cells were used for gene set enrichment analysis (GSEA) (Suppl. Tab. 3). Since PPAR agonist treatment might induce the expression of proteins not detectable under basal conditions in each cell line, qualitative Venn diagrams were first prepared after stringent filtering for at least 2 valid values in each cell type (Suppl. Tab. 4 S4_01, 22RV1 and Suppl. Tab. 5 S5_01, PC3). For pairwise statistical comparisons, we filtered the dataset for 70% completeness in 22RV1 and PC3 cells separately, imputed missing values by the BayesianRidge algorithm in InstantClue, and performed unpaired Welch T-tests with permutation-based FDR control enabled (*q*-value < 0.05). Results are summarized in corresponding data matrixes for 22RV1 (Suppl. Tab. 4 S4_02 and S4_03) or PC3 (Suppl. Tab. 5 S5_02 and S5_03). Volcano plots of pairwise comparisons were generated in Instant Clue with proteins meeting the fold change cut-off criteria of 1 color-coded and Venn Diagrams of overlapping significant proteins prepared as described above. All raw data were deposited to the ProteomeXchange Consortium via the PRIDE partner repository with the dataset identifier PXD060526 [48].

### Gene Set Enrichment Analysis (GSEA)

Gene Set Enrichment Analysis (GSEA) was performed in R (version 4.2.0) using the fgsea R package (version 1.22.0) [37]. For every gene, a ranking feature was calculated as a signed log_2_ fold change multiplied by log_10_ *p*-value. Genes were then sorted based on the ranking feature, and the sorted list was used as an input for GSEA. Hallmark gene sets, KEGG gene sets, and WikiPathways gene sets used for the analysis were downloaded using the msigdbr R package (version 7.5.1) from the MSigDB database (Suppl. Tab. 3) [39, 40].

### Seahorse Assay

22RV1 cells were adjusted to a density of 2 * 10^4^ cells, and PC3 cells to 9 * 10^3^ cells per 180 µl/well 24 h before measurement. Cells were treated with either 100 µM or 50 µM of the PPAR agonists Bezafibrate, Tesaglitazar, Pioglitazone, or the vehicle control (0.2% DMSO). On the assay day, media in the wells was replaced with Seahorse XF RPMI medium (pH = 7.4, 5 mM HEPES), containing the same concentrations of each PPAR agonist. The medium was supplemented with 1 mM pyruvate, 2 mM glutamine, and 10 mM glucose for the Mito Stress Test. The Glycolysis Stress Test included only 2 mM glutamine. Cells were washed once and equilibrated in a non-CO_2_ incubator for 45 min. The Mito Stress Test and Glycolysis Stress Test were performed as previously published by Pencik et al., 2023, and Klein et al., 2021, respectively [17, 49]. These tests measured Oxygen Consumption Rates (OCR) and Extracellular Acidification Rates (ECAR) using the Seahorse XF- 96 analyzer. Pharmacological agents Oligomycin, FCCP, Rotenone, and Antimycin A were prepared as 2.5 mM stock solutions in DMSO and diluted in Seahorse XF RPMI Medium before the assay. Glucose and 2-deoxy-D-glucose were dissolved in the medium on the assay day. In the Mito Stress Test, cells were treated with 1.5 µM Oligomycin, followed by 2 µM FCCP after 15 min, and then 1 µM Rotenone and Antimycin A for 15 min. The Glycolysis Stress Test involved treating cells with 11 mM glucose, 1.5 µM Oligomycin after 15 min, and 50 mM 2-deoxy-D-glucose. ATP production calculation was based on quantifying the OCR associated with the decline upon Oligomycin-induced blockade of mitochondrial ATP synthase, normalized to the baseline respiration. Glycolysis was assessed by measuring the ECAR upon adding 11 mM glucose to cells starved of glucose and pyruvate for 75 min, compared to baseline ECAR. Total protein content was used for normalization. Cells were seeded in 3 to 4 technical replicates, and the experiment was repeated three times. One-way ANOVA was used to evaluate statistical significance (ns = not significant *p* > 0.05, * *p* ≤ 0.05, ** *p* ≤ 0.01, *** *p* ≤ 0.001, **** *p* ≤ 0.0001). The graphs were plotted in GraphPad Prism v8.

### In vitro radiotracer cell uptake assay

90 µl of a cell suspension containing 2 * 10^5^ 22RV1 or PC3 cells were incubated in 96-well filter plates (MADVN6550, Merck Millipore, Darmstadt, Germany) with 60 µL of the respective radiotracer solution ([^18^F]FDG/[^18^F]FTHA: 0.4 MBq/mL, [^11^C]acetate: 2 MBq/mL). All three radiotracers were freshly produced on-site before the experiment. [^18^F]FDG uptake measurements were conducted in a glucose-free medium; [^18^F]FTHA and [^11^C]acetate uptake assays were conducted in the respective cell culture media. The filter plates were incubated with the radiotracer alone to assess unspecific binding. After 60 min of incubation at 37 °C, the cells were washed by vacuum filtration with 1 × PBS (2 × 200 µL) through the plate. The filters were transferred into tubes using a commercial punch kit and measured in a gamma counter (Wizard 2, PerkinElmer, Waltham, MA, USA). Radiotracer uptake was quantified as the percentage of added radioactivity. One-way ANOVA was used to evaluate statistical significance (ns = not significant *p* > 0.05, * *p* ≤ 0.05, ** *p* ≤ 0.01, *** *p* ≤ 0.001, **** *p* ≤ 0.0001). The graphs were plotted in GraphPad Prism v8.

### Untargeted NMR-based metabolomics

For untargeted metabolomics analysis, 2 * 10^6^ cells of 22RV1 and PC3 were seeded in 6 biological replicates in RPMI full media. After cell attachment overnight, the cells were treated with vehicle control (0.2% DMSO) and 100 µM of each PPAR agonist Bezafibrate, Tesaglitazar, and Pioglitazone for 24 h. Thereafter, the treated cells were scraped in the supernatant, centrifuged for 5 min at 4 °C, and washed three times with 1 × PBS. Each biological replicate was counted, equal cell numbers were centrifuged, and the pellet was lysed in 100% methanol. The samples were vortexed and snap-frozen in liquid nitrogen. This step was repeated 3 times, and the cell suspension was incubated at − 20°C for 1 h. After centrifugation, the supernatant samples were collected and dried in a speed vacuum concentrator overnight at 30 °C. The samples were then dissolved in a mixture containing phosphate buffer (520 µL, 0.135 mol/L), D2O (50 µL), and TSP (30 µL, 5.8 mmol/L) (Cambridge Isotope Laboratories) as internal standard and were analyzed by NMR. The NMR measurements were performed using a Bruker spectrometer operating at 600 MHz equipped with a cryogenically cooled probe and autosampler. 1H NMR spectra were obtained using the zgesgp pulse sequence at 25 °C, with 128 scans and 65,536 data points over a spectral width of 17,942.58 Hz [50]. Acquisition time was 1.82 s, and relaxation delay was 4.0 s. The NMR spectra were processed using Bruker Topspin 1.3 software, were Fourier-transformed after multiplication by line broadening of 0.3 Hz, and referenced to TSP at 0.0 ppm. The spectral phase and baseline were manually corrected. Each NMR spectrum was integrated using NMR Suite 10.0 profiler (ChenomX Inc, Edmonton, Canada) into 0.01 ppm integral regions (buckets) between 0.50 and 9.00 ppm, in which areas between 4.40 and 5.76 ppm containing residual water were removed. Each spectral region was normalized to the total spectral intensity to account for the different number of cells extracted for each sample. When a bucket was found to be discriminative in multivariate data analysis (see below), its corresponding NMR single was identified using the NMR Suite 10.0 library (ChenomX Inc, Edmonton, Canada). Multivariate data analysis, i.e. principal component analysis (PCA) and partial least-squares-discriminant analysis (PLS-DA), were performed using SIMCA-P + 17.0 software (Umetrics, Umeå, Sweden) as described previously [51]. PCA and PLS-DA models were fitted using bucketed NMR spectral data. In PLS-DA models fitted using bucketed spectral data, the variable importance for projection (VIP) of the spectral regions (bucket) was used to determine the discriminative spectral areas along the first two components. Spectral regions with VIP ≥ 1 and VIP minus its corresponding jackknife-based 95% confidence interval (CI) equal to or larger than 0.5 (VIP–CI ≥ 0.5) were considered discriminative. The model validity was investigated using permutation plots in SIMCA (Suppl. Tab. 6). Univariate analysis of selected metabolites was performed using the intensity of each signal divided by the total intensity of all signals (relative intensity). One-way ANOVA was used to evaluate statistical significance (ns = not significant *p* > 0.05, * *p* ≤ 0.05, ** *p* ≤ 0.01, *** *p* ≤ 0.001, **** *p* ≤ 0.0001).

### Bodipy lipid droplet staining

Intracellular neutral lipid droplets were stained using Bodipy 493/503. 22RV1 and PC3 cells (3 * 10^5^ cells/well) were seeded and treated with 100 µM of each PPAR agonist Bezafibrate, Tesaglitazar, and Pioglitazone for 24 h at 37 °C and 5% CO_2_. The cells were trypsinized, washed with 1 × PBS, and stained with 2.5 µM Bodipy staining solution in 1 × PBS. The staining was performed at 37 °C for 15 min, and DAPI was used to stain the nuclei. The Bodipy signal was acquired in the FITC channel for flow cytometry analysis, while the Pacific blue channel was used for the DAPI signal. The FACS data were acquired with the BD FACS Canto 2 (BD Biosciences) and analyzed with the FlowJo_V10 software and GraphPad Prism v8. One-way ANOVA was used to evaluate statistical significance (ns = not significant *p* > 0.05, * *p* ≤ 0.05, ** *p* ≤ 0.01, *** *p* ≤ 0.001, **** *p* ≤ 0.0001).

### Immunofluorescence (IF) staining on cells

Cells were seeded onto superfrost microscopy slides and cultured for 24 h in a humidity chamber under standard culture conditions. After attachment onto the slides, the cells were washed three times in 1 × PBS, fixed in 4% PBS-buffered formaldehyde at RT for 15 min, rewashed three times with 1 × PBS, dried, and frozen at − 20 °C. On the day of staining, the frozen cells were thawed at RT for 30 min, followed by washing three times with 1 × PBS + 0.2% Tween20. After that, the slides were covered with a Universal block for 7 min and washed with 1 × PBS + 0.2% Tween20. The primary antibody in 1 × PBS + 1% BSA was incubated at 4 °C overnight and then washed with 1 × PBS + 0.2% Tween20 before the cells were incubated with anti-rabbit secondary antibody labelled with Alexa Fluor 488 in 1 × PBS + 1% BSA at RT for 30 min. The stained cells were washed three times with 1 × PBS + 0.2% Tween20, stained with DAPI 1:50,000 in distilled H_2_O at RT for 10 min, washed three times, and mounted with fluorescence mounting media. The staining was acquired with the Axio Imager M2 (Zeiss) using the Zen2 blue edition software (ZEISS).

### Scratch assay

The scratch assay was performed with the Incucyte S3 (Sartorius) live cell imaging device. 6 * 10^4^ 22RV1 cells/well and 2 * 10^4^ PC3 cells/well were seeded into Imagelock 96-well plates in RPMI full media to achieve a confluent monolayer. After cell attachment, the cells were serum-starved in RPMI + 5% fat-free BSA + 1% Pen/Strep for 16 h. Thereafter, the cells were treated with 100 µM of each PPAR agonist Bezafibrate, Tesaglitazar, Pioglitazone, or the vehicle control (0.2% DMSO) in serum-starved media for 24 h. The “Incucyte Woundmaker” was used and handled according to product instructions to create a wound in each well. Cell migration was assessed at 37 °C and 5% CO_2_ for 24 h by live-cell imaging and image acquisition with an interval of 1 h. The Incucyte basic analysis software was used for data analysis. One-way ANOVA was used to evaluate statistical significance (ns = not significant *p* > 0.05, * *p* ≤ 0.05, ** *p* ≤ 0.01, *** *p* ≤ 0.001, **** *p* ≤ 0.0001). The graphs were plotted in GraphPad Prism v8.

### Xenograft

NSG mice were obtained from Charles River and housed in groups of 2–5 mice per cage in individually ventilated cages with a 12-light/dark cycle. All procedures were carried out under UK Home Office license PP0918061 according to the Animals (Scientific Procedures) Act 1986 and were approved by the University of Cambridge Animal Welfare and Ethical Review Board (AWERB). 22RV1 and PC3 cells were cultured in RPMI supplemented with 10% FBS + 1% Pen/Strep until they reached the number necessary for injecting the mice. 2 million cells were injected per mouse after being suspended in Matrigel (Corning) diluted 1:2 with PBS, into the left flank of NSG male mice at average 8 weeks of age. Tumors were measured daily with manual callipers, and tumor volumes were estimated using the modified ellipsoid formula: V = ab^2^/2, where a and b (a > b) are length and width measurements, respectively. Once tumors reached ~ 200 mm^3^, mice were randomly allocated into treatment groups (*n* = 6 mice per group) and treated daily with the following agents by oral gavage at 10 µL/g body weight: vehicle (20% hydroxypropyl-beta cyclodextrin), Tesaglitazar (0.4 mg/kg), and Pioglitazone (10 mg/kg) for 14 days. Mice were euthanized at the end of the 14 days of treatment or once tumors reached 15 mm in any direction. The maximal tumor size permitted by our Project Licence (20 mm) was not exceeded in any of the studies. Tumors were fixed in 10% neutral-buffered formalin for 24 h before changing to 70% ethanol and paraffin embedding.

### Immunohistochemistry

Immunohistochemistry (IHC) stainings were performed on formalin-fixed and paraffin-embedded (FFPE) mouse tissue sections according to standard protocol. The antibodies used are listed in Tab. 1. All stained sections were scanned with the Olympus VS200 Scanner and quantified via the Qupath- 0.5.1 software.

### Patient cohort

After approval by the local ethics committee of Medical University Innsbruck (Vote number: 1301/2023), a retrospective data analysis based on medical records from men with local PCa who received radical prostatectomy (RP) between 2014 and 2023 was conducted. In total, data on 69 patients was obtained; 49 had T2D, and another 20 patients without diabetes were used as a comparison group. Only 3 of the patients were found to be treated with PPAR agonist, 15 patients were using SGLT2 inhibitor, 17 patients were using Metformin alone, and another 14 patients were treated with Metformin in combination with DDP4. Only 2 patients had T2D without medication, and 4 used insulin alone. The median age in the T2D group was 76 years, and 72 years in the control group. The following parameters were collected and analyzed to evaluate differences between the two cohorts: age, body mass index (BMI), initial PSA at diagnosis, Gleason score (GS) at biopsy and RP, TNM classification, status of resection margins according to the UICC classification, biochemical recurrence (BCR) and status of PSA persistence. KM curves comparing the different patient groups were performed in the GraphPad Prism v8 software using the log-rank (Mantel-Cox) test with *p* ≤ 0.05.

## Results

### PCa patients with high *PPARG* expression have reduced survival probabilities

**Fig. 1:**
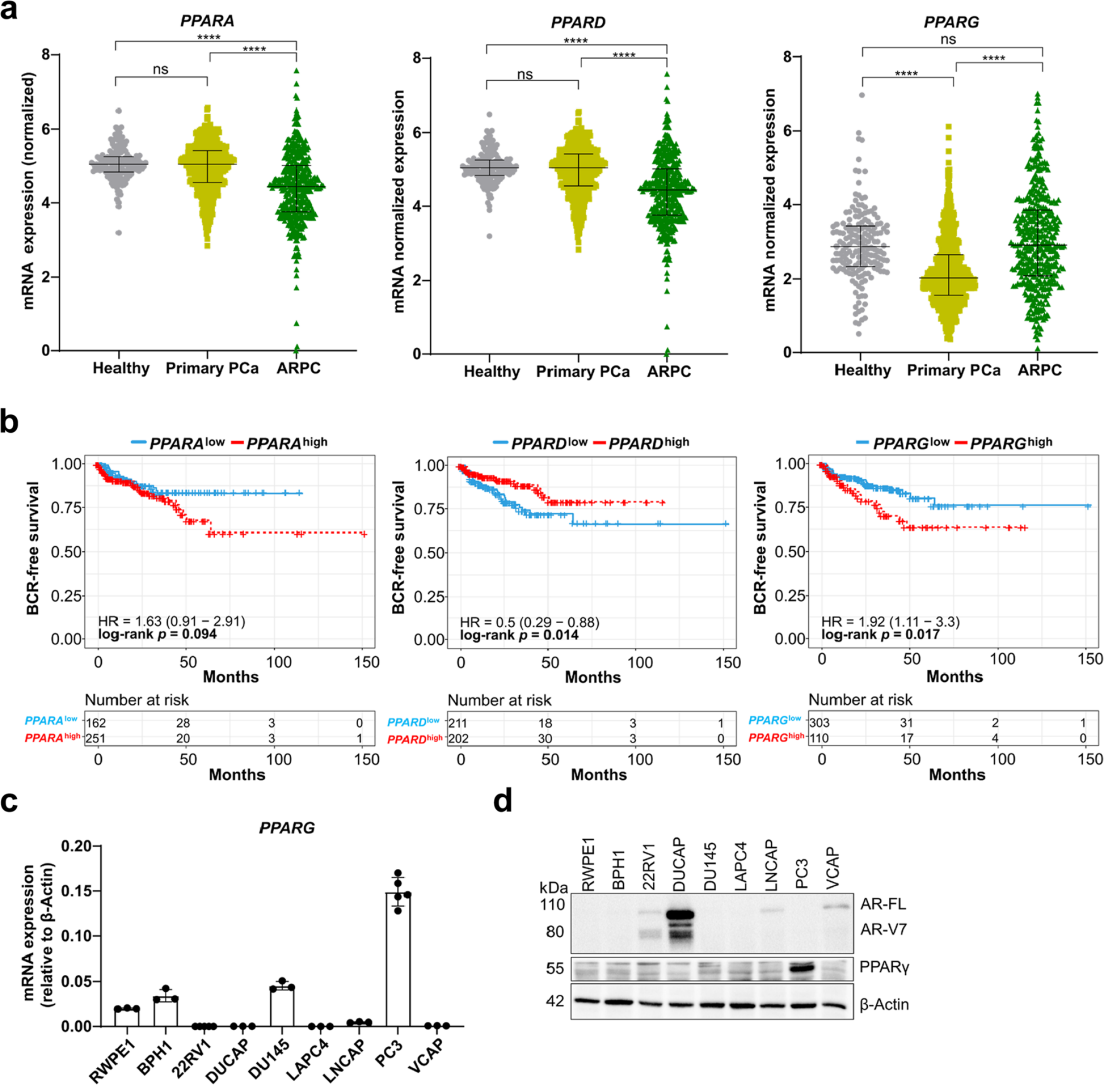
PCa patients with high *PPARG* expression have reduced survival probabilities. **a** Normalized mRNA expression of *PPARA* (left), *PPARD* (middle), and *PPARG* (right) based on bulk RNA-sequencing data from the Prostate Cancer Atlas comprising healthy (*n* = 173), primary PCa (*n* = 708) and ARPC patients (n = 428). Significance was tested via one-way ANOVA (ns = not significant *p *> 0.05, * *p *≤ 0.05, ** *p *≤ 0.01, *** *p* ≤ 0.001, **** *p *≤ 0.0001). **b** Kaplan-Meier analysis showing BCR-free survival for primary PCa patients (*n* = 333) from the TCGA-PRAD cohort comparing high versus low expression of *PPARA* (left), *PPARD* (middle), and *PPARG* (right). Significance was determined using Cox regression analysis (*p *≤ 0.05). **c** QRT-PCR analysis of basal mRNA levels of *PPARG* relative to β-Actin in indicated PCa cell lines. Data are representative of the means ± standard deviations (SD) of biological triplicates. **d** Western Blot analysis of androgen receptor full-length (AR-FL) and the splice variant 7 (AR-V7), as well as PPARγ in different PCa cell lines. Β-Actin was used as a loading control

We explored the role of *PPARA*, *PPARD*, and *PPARG* at different stages of PCa in the Prostate Cancer Atlas, which comprises normalized mRNA expression levels of gene transcripts in various patient cohorts. Patients with AR pathway-independent PCa (ARPC) (*n* = 428) showed significantly reduced expression of *PPARA* and *PPARD* compared to healthy (*n* = 173) and primary PCa patients (*n* = 708) (Fig. 1a). However, *PPARG* expression in primary PCa patients was significantly lower compared to healthy patients but was increased in ARPC (Fig. 1a). Further analysis of TCGA-PRAD data (*n* = 333 patients) revealed that high *PPARG* expression correlated with a significantly lower biochemical recurrence (BCR)-free survival (Fig. 1b). In contrast, *PPARA* expression did not affect survival, while high *PPARD* expression was linked to improved survival outcomes in PCa patients. Consequently, we assessed whether the adverse survival outcomes associated with high *PPARG* expression were influenced by tumor suppressors of PCa such as phosphatase and tensin homolog (*PTEN*) and signal transducer and activator of transcription 3 (*STAT3*). We discovered that the reduced survival probability of patients with high *PPARG* expression was not *PTEN*-dependent. However, the concurrent loss of *STAT3* was strongly associated with a reduced survival probability (Suppl. Figure 1a, b). Additionally, we examined *PPAR* and *AR* expression across a spectrum of PCa cell lines, from healthy prostate tissue to metastatic stages, at both mRNA and protein levels. We observed low *PPARA*, *PPARD*, and *PPARG* mRNA expression across all the screened PCa cell lines. However, *PPARG* was highly expressed at mRNA and protein levels in the metastatic PC3 cells, representing ARPC patients (Fig. 1c, d, Suppl. Figure 1c, d). Thus, for our mechanistic study, we selected the two contrasting cell lines 22RV1 and PC3. The 22RV1 cells originate from primary PCa and are derived from a xenograft mouse model (described as primary PCa cells from this section on), and are positive for AR full-length (AR-FL) and AR splice variant 7 (AR-V7) but negative for PPARγ. The bone marrow metastases PC3 cells are negative for AR but positive for PPARγ.

### PPAR agonists inhibit cell proliferation in primary and metastatic PCa cells

**Fig. 2:**
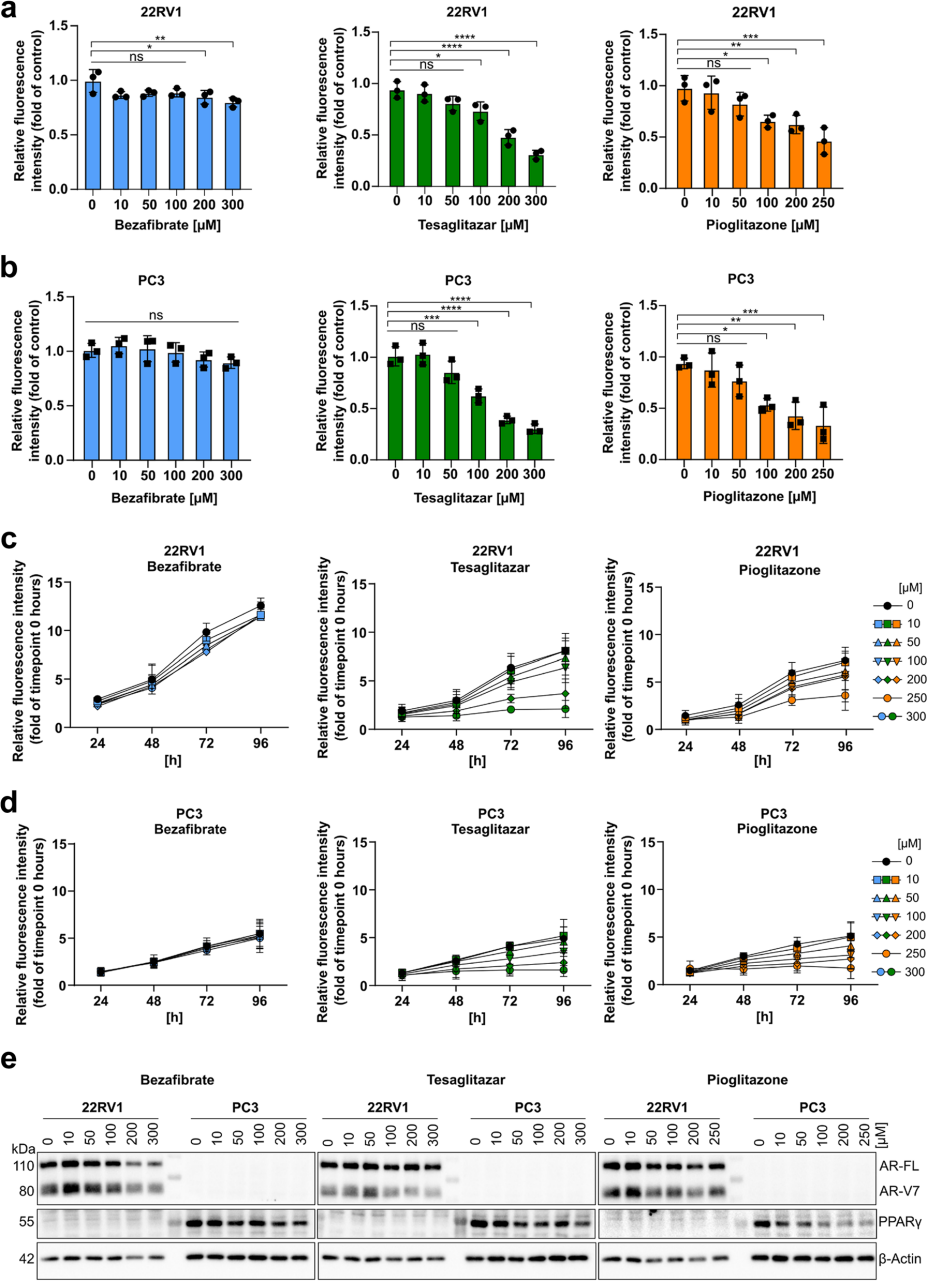
PPAR agonists inhibit cell proliferation in primary 22RV1 and metastatic PC3 cells. **a, b** Relative fluorescence intensities normalized to vehicle control (0.2 % DMSO) of resazurin-based metabolic activity assay of 22RV1 (**a**) and PC3 cells (**b**) treated with serial dilutions of the PPAR agonists Bezafibrate, Tesaglitazar, and Pioglitazone for 72 hours. Significance was determined by one-way ANOVA (ns = not significant *p *> 0.05, * *p *≤ 0.05, ** *p *≤ 0.01, *** *p *≤ 0.001, **** *p *≤ 0.0001). **c, d** Relative fluorescence intensities normalized to time point 0 hours of resazurin-based metabolic activity assay of 22RV1 (**c**) and PC3 cells (**d**) following treatment with PPAR agonists or control (0.2 % DMSO) at indicated concentrations (highest concentration for Pioglitazone was 250 µM) in time-course experiments (24, 48, 72, and 96 hours). Data of resazurin assays are representative of the means ± SD of biological triplicates. **e** Western blot showing AR-FL, AR-V7, and PPARγ protein levels after 24 hours of treatment with increasing concentrations of Bezafibrate (left), Tesaglitazar (middle), and Pioglitazone (right). Β-Actin was used as a loading control. Representative western blots of biological triplicates are shown

To assess the effect of PPAR agonists on PCa cell viability, we measured cellular metabolic activity indicated by their redox potential via the resazurin assay. Our results showed that the dual PPARα/γ agonist Tesaglitazar and the PPARγ agonist Pioglitazone significantly decreased cell viability in a dose-dependent manner after 72 h of exposure for both 22RV1 and PC3 cells compared to the vehicle-treated control cells. The PPARα agonist Bezafibrate did not influence cell viability in any cell line (Fig. 2a, b). Moreover, dose- and time-dependent analyses from 24 to 96 h of exposure to Tesaglitazar and Pioglitazone revealed reduced metabolic activity, which can indirectly be associated with cell proliferation, while Bezafibrate showed no effect in any cell line (Fig. 2c, d). In addition, by employing the CyQuant assay to quantify cellular DNA content after 72 h of treatment with PPAR agonists, we discovered that Tesaglitazar and Pioglitazone markedly reduced cellular DNA content in both the 22RV1 and PC3 cells, while Bezafibrate showed no impact (Suppl. Figure 2a, b). Furthermore, Western blot analysis performed 24 h post-PPAR agonist treatment indicated that PPARγ protein levels were reduced in PC3 cells with increasing concentrations of Tesaglitazar and Pioglitazone. However, AR expression remained mainly unaffected in 22RV1 cells (Fig. 2e, and quantitative densitometric analysis in Suppl. Figure 2c). Moreover, flow cytometry analysis revealed that treatment with PPAR agonists for 24 to 72 h did not induce apoptosis in either of the cell lines (Suppl. Figure 2 d, e).

### Proteome analysis reveals altered metabolic pathways in primary 22RV1 and metastatic PC3 cells

**Fig. 3:**
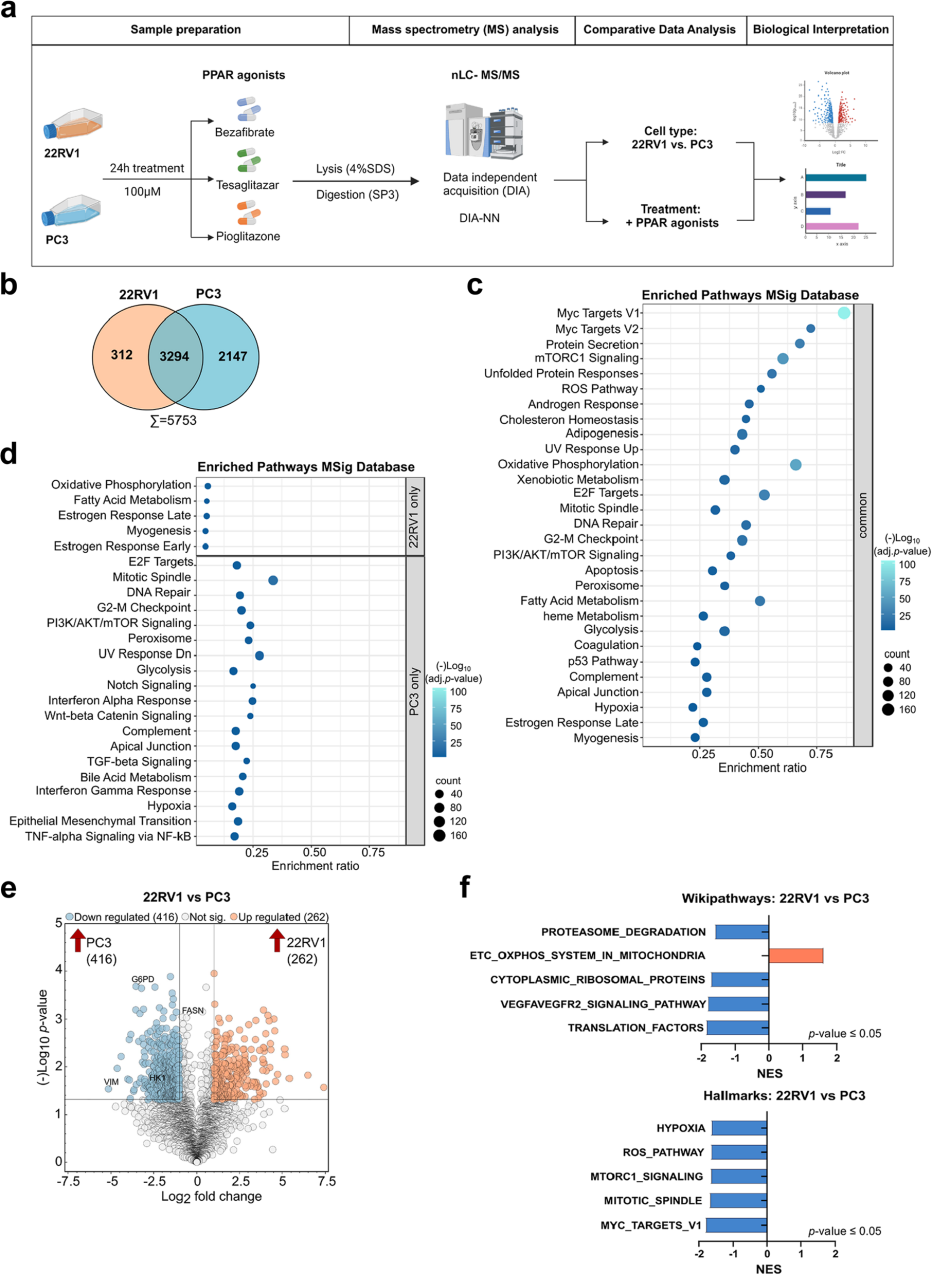
Proteome analysis reveals altered metabolic pathways in primary 22RV1 and metastatic PC3 cells. **a** Workflow of sample preparation and comparative proteome analysis of 22RV1 and PC3 cells (”Cell type”) under basal conditions or following treatment with PPAR agonists (24 hours, 100 µM, vehicle control = 0.2 % DMSO) as indicated. **b** Qualitative Venn diagram showing unique (“only”) and common protein identities in 22RV1 (orange) and PC3 (blue) cells under basal (untreated) conditions. **c, d** MSig database enrichment analysis for pathways of proteins that were detected in both cell lines (**c**) and in 22RV1 or PC3 cells only (**d**). **e** Volcano plot showing differences in protein expression levels (log_2_ fold change) among 22RV1 and PC3 cells under basal culture conditions with -log_10_ *p*-values (colored dots indicate proteins with a log_2_ fold change = ≤ -1|≥ 1 and -log_10_ *p* ≥ 1.3 assessed by unpaired Welch´s T-test with BJH correction (FDR = 0.05)). **f** GSEA analysis of significantly deregulated pathways (nested enrichment score (NES) = ≤ -1|≥ 1, *p *≤ 0.05) comparing 22RV1 with PC3 cells under basal conditions is based on the Wikipathways and HALLMARKS database

To get an unbiased view of the proteome landscape of primary and metastatic PCa cells (represented by 22RV1 and PC3, respectively), we analyzed their proteome under basal culture conditions and following treatment with PPAR agonists Bezafibrate, Tesaglitazar, and Pioglitazone. Both cell lines were subjected to 100 µM of each PPAR agonist for 24 h, assuming that early proteome alterations might provide mechanistic insight into the effect of PPAR agonists on cell viability and proliferation. A schematic outline of the analysis workflow is given in Fig. 3a. Our initial analysis focused on qualitatively comparing the basal proteomic profiles of 22RV1 and PC3 cells. We identified a set of 3294 proteins commonly detected in both cell lines, alongside 312 proteins unique to 22RV1 cells and 2147 proteins unique to PC3 cells (Fig. 3b, Suppl. Tab. 1). Enrichment analysis of the shared proteome revealed that both cell lines expressed proteins rich in pathways such as Myc targets, oxidative phosphorylation (OXPHOS), and mechanistic target of rapamycin kinase complex 1 (mTORC1) signaling (Fig. 3c, Suppl. Tab. 2). Analysis of the proteins identified only in 22RV1 cells showed enrichment of OXPHOS and fatty acid metabolism pathways. In contrast, the proteins identified only in PC3 cells were predominantly associated with epithelial to mesenchymal transition (EMT) and transforming growth factor (TGF)-beta signaling (Fig. 3d, Suppl. Tab. 2). Comparative statistical analysis of the 3294 shared proteins between 22RV1 and PC3 cells revealed more than twenty percent as being significantly differentially expressed (nominal *p* ≤ 0.05, log_2_ fold change ≤ − 1|≥ 1). We discovered 262 proteins with higher abundance in 22RV1 and 416 proteins with higher abundance in PC3 cells (Fig. 3e, Suppl. Tab. 1). Notably, proteins such as vimentin (VIM), glucose- 6-phosphate dehydrogenase (G6PD), and hexokinase 1 (HK1) showed higher expression levels in PC3 cells, while being downregulated in 22RV1 cells (Fig. 3e). Gene set enrichment analysis (GSEA) of these differentially expressed proteins (DEP), utilizing the Wikipathways and HALLMARKS databases, indicated that the “electron transport chain (ETC) OXPHOS system in mitochondria” pathway was upregulated in 22RV1 cells. Conversely, pathways associated with reactive oxygen species (ROS) production and mTORC1 signaling were downregulated in 22RV1 cells but upregulated in PC3 cells (Fig. 3f, Suppl. Tab. 3). These findings suggest distinct metabolic regulations in primary versus metastatic PCa cells, particularly in pathways such as OXPHOS, mTORC1, and fatty acid metabolism.

### PPAR agonists alter the expression of proteins involved in metabolic pathways in primary 22RV1 and metastatic PC3 cells

**Fig. 4:**
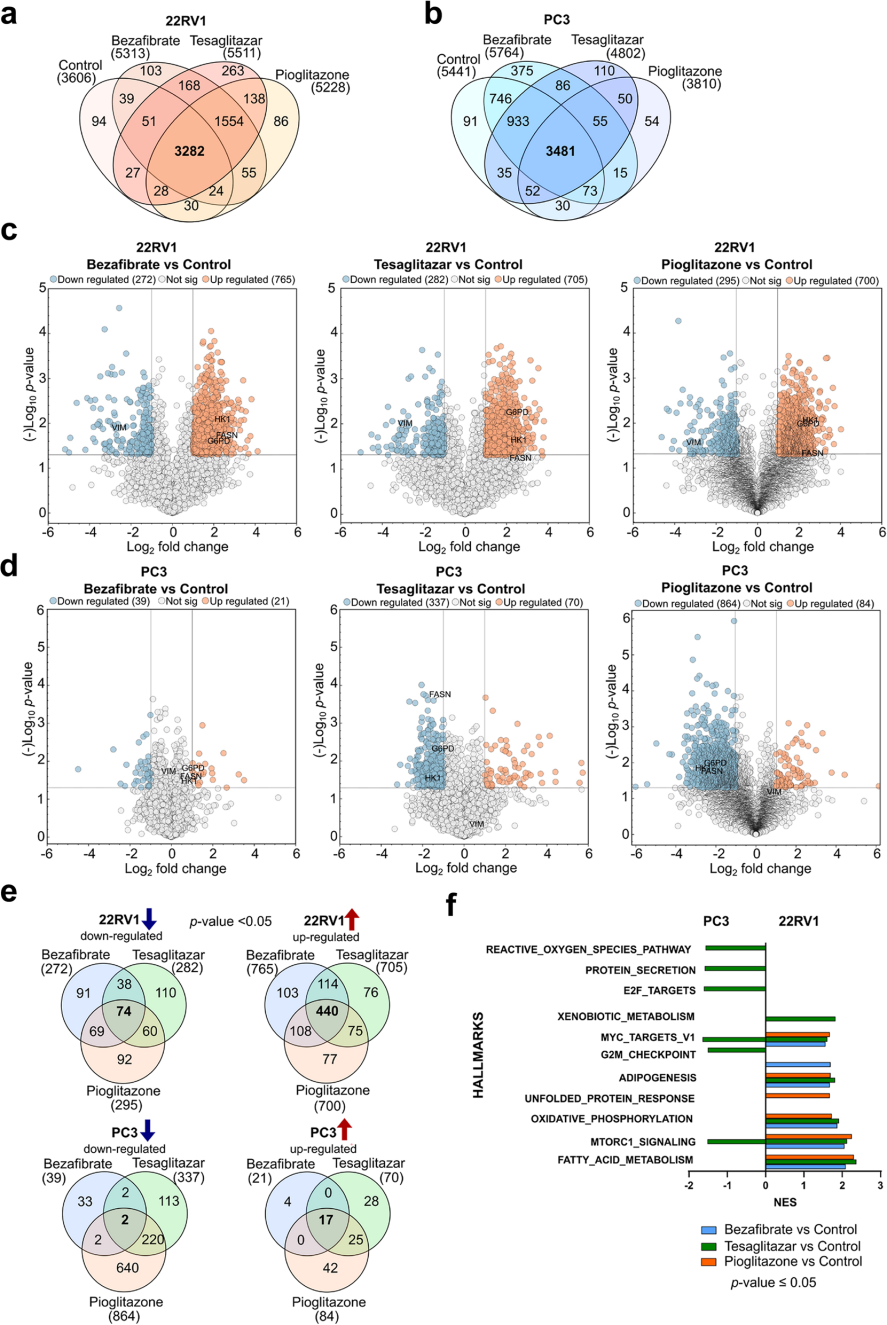
PPAR agonists alter the expression of proteins involved in metabolic pathways in primary 22RV1 and metastatic PC3 cells. **a, b** Qualitative Venn diagrams of proteins detected in 22RV1 (**a**) or PC3 cells (**b**) and after 24 hours of treatment with the PPAR agonists Bezafibrate, Tesaglitazar, and Pioglitazone (100 µM, vehicle control = 0.2 % DMSO). **c, d** Volcano plots of DEP comparing control samples of 22RV1 (**c**) or PC3 (**d**) with each PPAR agonist. **e **Venn diagrams of proteins that were commonly up - or downregulated by all PPAR agonists in 22RV1 or PC3 cells (log_2_ fold change = ≤ -1|≥ 1 and -log_10_ *p *≥ 1.3). Significance was assessed by an unpaired Welch´s T-test with BJH correction (FDR = 0.05). **f** GSEA analysis of significantly deregulated pathways (nested enrichment score (NES) = ≤ - 1|≥ 1, *p *≤ 0.05) in 22RV1 and PC3 cells after treatment with each PPAR agonist compared to the control sample based on the HALLMARKS database

Further investigation into the impact of PPAR agonists on the proteomic landscape of 22RV1 and PC3 cells revealed significant alterations. In stringent qualitative intersection comparisons of proteins identified two times under each condition, 3282 proteins were commonly detected in the vehicle and PPAR agonist-treated 22RV1 cells (Fig. 4a). In the PC3 cells, 3481 proteins were commonly detected (Fig. 4b). Additionally, proteins specific to each treatment condition were identified, indicating a distinct proteomic response to each drug in both cell lines (Fig. 4a, b). DEP analysis comparing the proteome of vehicle-treated with Bezafibrate, Tesaglitazar, and Pioglitazone treated 22RV1 cells revealed significant upregulation of fatty acid synthase (FASN), G6PD, and HK1 in response to PPAR agonist treatment. At the same time, proteins such as VIM were downregulated (*p* ≤ 0.05, log_2_ fold change ≤ − 1|≥ 1) (Fig. 4c, Suppl. Tab. 4). In PC3 cells, the impact of PPAR agonist treatment was much less pronounced and almost neglectable for Bezafibrate (Fig. 4d, Suppl. Tab. 5). Tesaglitazar and Pioglitazone treatment notably resulted in a significant downregulation of proteins including FASN, G6PD, and HK1 (*p* ≤ 0.05, log_2_ fold change ≤ − 1|≥ 1) (Fig. 4d). Compared to the vehicle-treated 22RV1 cells 74 proteins were significantly downregulated and 440 proteins were upregulated across all three PPAR agonist treatments. At the same time, drug-specific regulatory effects were observed (Fig. 4e, upper panel). In contrast, in the PC3 cells, only 2 proteins were significantly downregulated across all three PPAR agonist treatments compared to the vehicle-treated sample, and 17 proteins were significantly upregulated, in addition to the drug-specific regulatory effects (Fig. 4e, lower panel). GSEA was conducted to evaluate the impact of PPAR agonist treatment on metabolic and signaling pathways in both cell lines. The 22RV1 cells' most significantly upregulated pathways were Myc targets, adipogenesis, OXPHOS, mTORC1 signaling, and fatty acid metabolism, following treatment with each PPAR agonist, according to the HALLMARKS database. In contrast, PC3 cells exhibited significant downregulation in pathways such as ROS production, Myc targets v1, and mTORC1 signaling, particularly upon treatment with especially Tesaglitazar (Fig. 4f, Suppl. Tab. 3). Further analysis using GSEA identified leading-edge proteins associated with the mTORC1 pathway and glycolysis. Log_2_-transformed fold changes revealed that treatment with each PPAR agonist upregulated leading-edge proteins of the mTORC1 pathway, such as G6PD or glutathione-disulfide reductase (GSR) for 22RV1 cells. In contrast, these proteins were downregulated in PC3 cells upon treatment with Tesaglitazar and Pioglitazone (Suppl. Figure 3a). Furthermore, the basal protein levels of mTORC1 pathway-associated proteins were found to be lower in 22RV1 cells than in PC3 cells, indicating a differential baseline expression of these proteins between the cell lines (Suppl. Figure 3b). PPAR agonist treatment also led to an upregulation of glycolytic proteins such as malic enzyme 2 (ME2) or dihydrolipoamide dehydrogenase (DLD) in the 22RV1 cells. In contrast, Pioglitazone treatment resulted in the downregulation of these proteins in the PC3 cells (Suppl. Figure 3c). Log_2_ transformed basal abundances of glycolysis-associated leading-edge proteins showed higher basal levels in PC3 compared to the 22RV1 cells (Suppl. Figure 3 d).

### PPARγ agonist Pioglitazone reprograms primary and metastatic PCa cell metabolism and induces an epithelial phenotype in 22RV1 cells

**Fig. 5:**
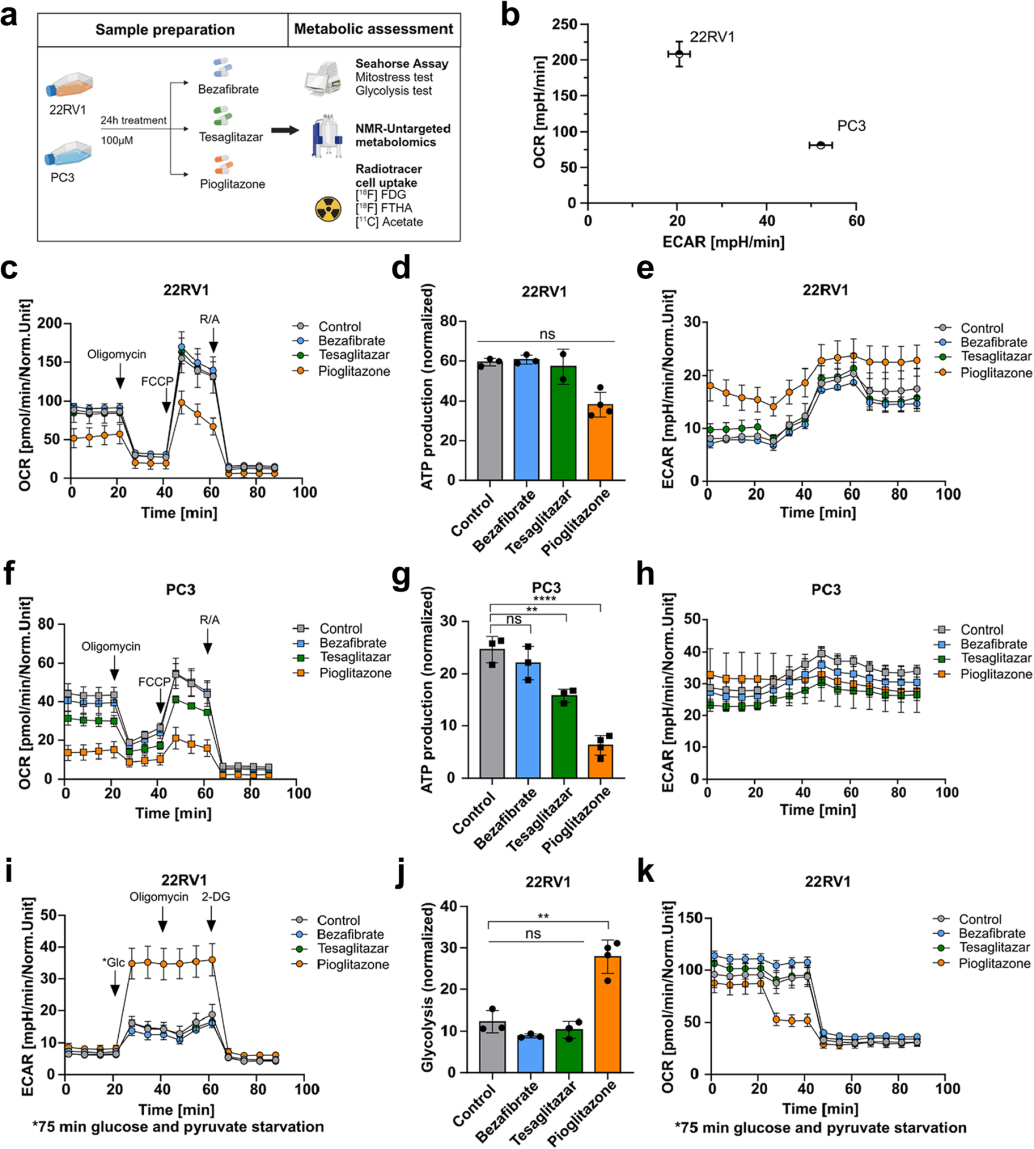

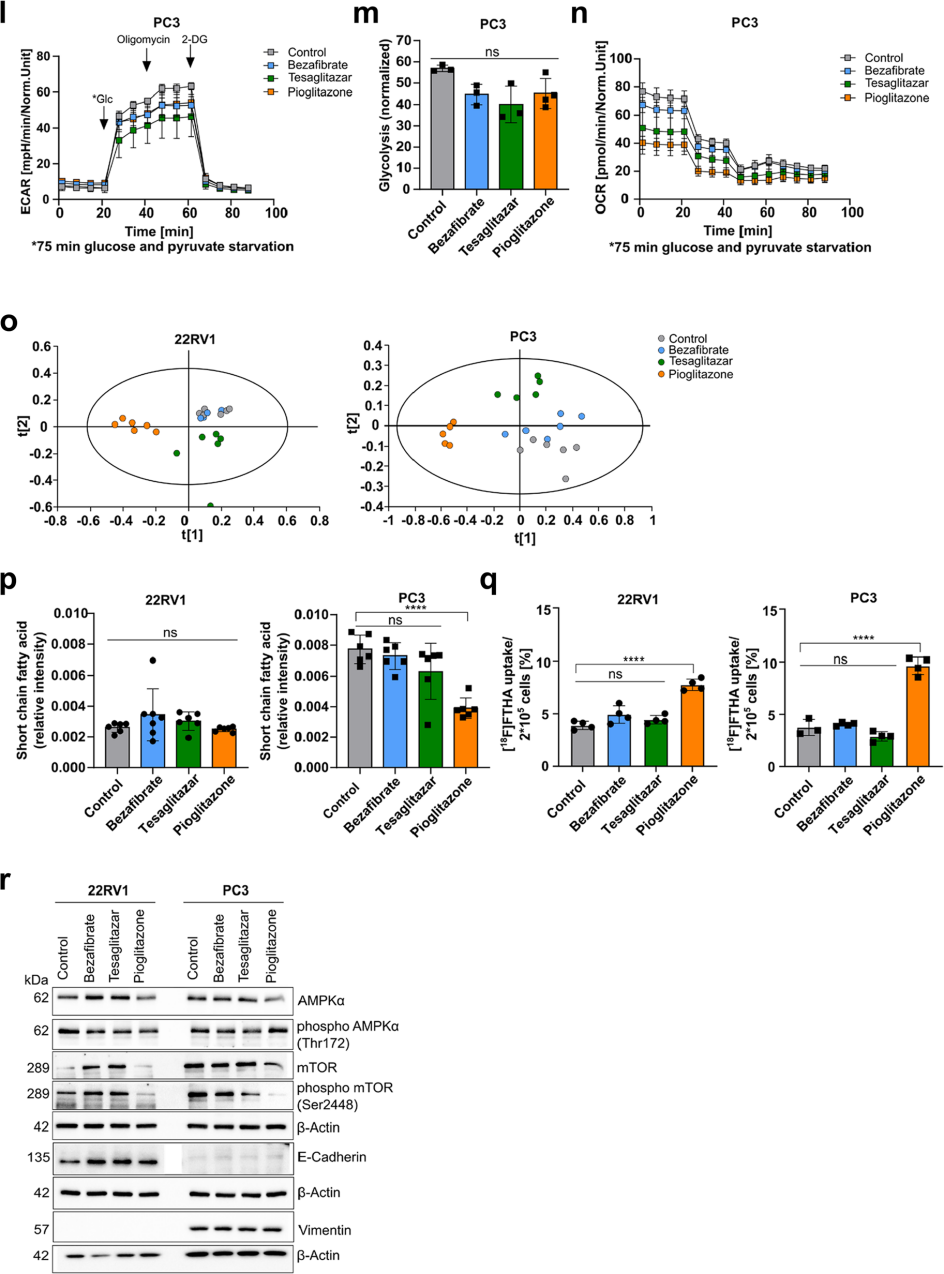
PPARγ agonist Pioglitazone reprograms primary and metastatic PCa cell metabolism and induces an epithelial phenotype in 22RV1 cells. **a** Workflow scheme for metabolic assessment based on the Seahorse assay, NRM untargeted metabolomics, and radiotracer cell uptake of 22RV1 and PC3 cells upon PPAR agonist Bezafibrate, Tesaglitazar, and Pioglitazone treatment (24 hours, 100 µM, vehicle control = 0.2 % DMSO). **b** Metabolic profile of 22RV1 and PC3 cells comparing basal oxygen consumption rate (OCR) and extracellular acidification rate (ECAR) calculated from fourth basal timepoint before Oligomycin injection. **c, d, e** Mitochondrial stress-induced OCR (**c**), ATP production (**d**) and ECAR in 22RV1 cells under control culture conditions and PPAR agonist treatment (**e**). Oligomycin was used as an inhibitor of ATP synthase/complex V, FCCP for maximal respiration by chemical uncoupling of the mitochondrial membrane gradient, and Rotenone Antimycin A (R/A) for non-mitochondrial respiration by complex I and III inhibition. **f, g, h **Mitochondrial stress-induced OCR responses (**f**), ATP production (**g**), and ECAR in PC3 cells (**h**) as described for 22RV1 cells. **i, j, k** Glycolysis test in 22RV1 after glucose and pyruvate starvation and treatment of PPAR agonists, as described above. Glucose injection after starvation serves as a measure for glycolysis, Oligomycin as the remaining contribution of OXPHOS to cellular energy production, and 2-deoxy-glucose (2-DG) for non-glycolytic ECAR contribution. ECAR profiles upon glucose injection following 75 minutes of glucose and pyruvate starvation (**i**), normalized glycolysis of 22RV1 cells treated with PPAR agonists (**j**), and normalized OCR (**k)**.** l, m, n** Glycolysis tests in PC3 cells as described for 22RV1 cells (**i, j, k)**. **o **Score plot of partial least squares-discriminant analysis (PLS-DA) model for bucketed NMR spectral data from 22RV1 (left; the model parameters for the two components fitted were as follows: R2Y = 0.498, Q2Y = 0.301) and PC3 cell extracts (right; the model parameters for the three components fitted were as follows: R2Y = 0.696, Q2Y = 0.499) treated with PPAR agonists as described above. **p** Relative abundance of short-chain fatty acids, resulting from the untargeted metabolomics of PPAR agonist treated 22RV1 (left) and PC3 cells. **q** [^18^F]FTHA uptake after 24 hours of treatment with each PPAR agonist in 22RV1 and PC3 cells. **r** Western Blot analysis of mTOR, phospho mTOR (Ser2448), AMPKα, phospho AMPKα (Thr172), E-Cadherin, and Vimentin expression in control and PPAR agonist treated 22RV1 and PC3 cells, β-Actin was used as a loading control. One representative experiment of the Seahorse assay profile is shown. Significance was evaluated by one-way ANOVA (ns = not significant *p *> 0.05, * *p *≤ 0.05, ** *p *≤ 0.01, *** *p *≤ 0.001, **** *p *≤ 0.0001). Data are representative of the means ± SD biological triplicates

We explored the metabolic effect of each PPAR agonist (24 h treatment, 100 µM) on the 22RV1 and PC3 cells using Seahorse Assay-based metabolic profiling, untargeted metabolomics via nuclear magnetic resonance (NMR) spectroscopy, and radiotracer cell uptake assays (Fig. 5a). Comparisons of basal oxygen consumption rate (OCR) and extracellular acidification rate (ECAR) from a mitochondrial stress test revealed an increased OCR in 22RV1 cells, indicating their reliance on OXPHOS. Conversely, PC3 cells exhibited a higher ECAR, suggesting a greater dependency on glycolysis (Fig. 5b). The treatment with Pioglitazone at 50 µM or 100 µM of 22RV1 cells resulted in a decreased basal and ATP synthase-linked OCR indicative of reduced mitochondrial ATP production, in line with an increase in ECAR, indicative of a metabolic shift towards glycolysis (Fig. 5c-e, Suppl. Figure 4a). In PC3 cells both Tesaglitazar and Pioglitazone decreased basal and ATP synthase-linked OCR, without altering ECAR (Fig. 5f-h, Suppl. Figure 4b). In line with the latter data, a glycolysis stress test, following glucose and pyruvate deprivation for 75 min, demonstrated that Pioglitazone treatment increased ECAR and glycolytic activity and decreased OCR in 22RV1 cells (Fig. 5i-k, Suppl. Fiure 4c). In the PC3 cells the glycolysis test resulted in a slight but insignificant reduction of ECAR and glycolysis after PPAR agonist treatment, with Tesaglitazar and Pioglitazone also reducing OCR (Fig. 5l-n, Suppl. Figre 4d). To underpin these findings, we measured [^18^F]fluoro-2-deoxy-D-glucose ([^18^F]FDG) uptake in 22RV1 and PC3 cells. Baseline measurements showed significantly increased [^18^F]FDG uptake in PC3 cells compared to 22RV1 cells, supporting our earlier results in the Seahorse assay (Suppl. Figure 4e). Furthermore, Pioglitazone treatment resulted in an increased [^18^F]FDG uptake trend in the 22RV1 cells (*p* = 0.1411), while PC3 cells were unaffected (Suppl. Figure 4f). To expand our understanding of the metabolic alterations induced by PPAR agonists, we conducted NMR-based untargeted metabolomics of the PPAR agonist-treated 22RV1 and PC3 cells. Applying the partial least squares-discriminant analysis (PLS-DA) model, Pioglitazone-treated samples were distinctly separated from the vehicle-treated and Bezafibrate and Tesaglitazar-treated samples along the horizontal axis, highlighting a considerable effect on the metabolome of both cell lines. However, Tesaglitazar treatment primarily led to a separation from the vehicle-treated control on the vertical axis, while Bezafibrate treatment did not result in any clear separation from the vehicle-treated control (Fig. 5o, Suppl. Tab. 6). Further analysis revealed a significant reduction in short-chain fatty acids upon Pioglitazone treatment in PC3 cells, but no such change was observed in 22RV1 cells (Fig. 5p). To determine if more fatty acids are taken up by the cells when they are treated with PPAR agonists, we performed [^18^F]fluoro-6-thia-heptadecanoic acid ([^18^F]FTHA) and [^11^C]acetate tracer uptake assays. Especially for the PC3 cells, a significant increase in uptake of both tracers was observed (Fig. 5q, Suppl. Figure 4 g). Despite this increased fatty acid uptake, there was a reduction in lipid droplets in the PC3 cell line (Suppl. Figure 4 h). At the molecular level, mTOR and 5'AMP-activated protein kinase alpha (AMPKα) are considered the central regulators of metabolic stress and FAO in cancer cells, due to their phosphorylation and activation via the PI3K/Akt and AMPK/LKB1 signalling pathway respectively. Western blot analysis of PPAR agonists treated 22RV1 and PC3 cells revealed a reduction of activated phospho AMPKα (Thr172) only in the 22RV1 cells. However, Pioglitazone decreased the levels of phospho mTOR (Ser2448) in both cell lines (Fig. 5r, quantitative densitometric analysis in Suppl. Figure 4i). Importantly, this metabolic reprogramming co-occurred with upregulation of epithelial marker E-Cadherin in PPAR agonist-treated 22RV1 cells. In contrast, the mesenchymal marker VIM remained unchanged in the PC3 cells (Fig. 5r, Suppl. Figure 4i, j).

### Pioglitazone suppresses cell migration of PCa cells and growth of metastatic PC3 xenograft tumors

**Fig. 6:**
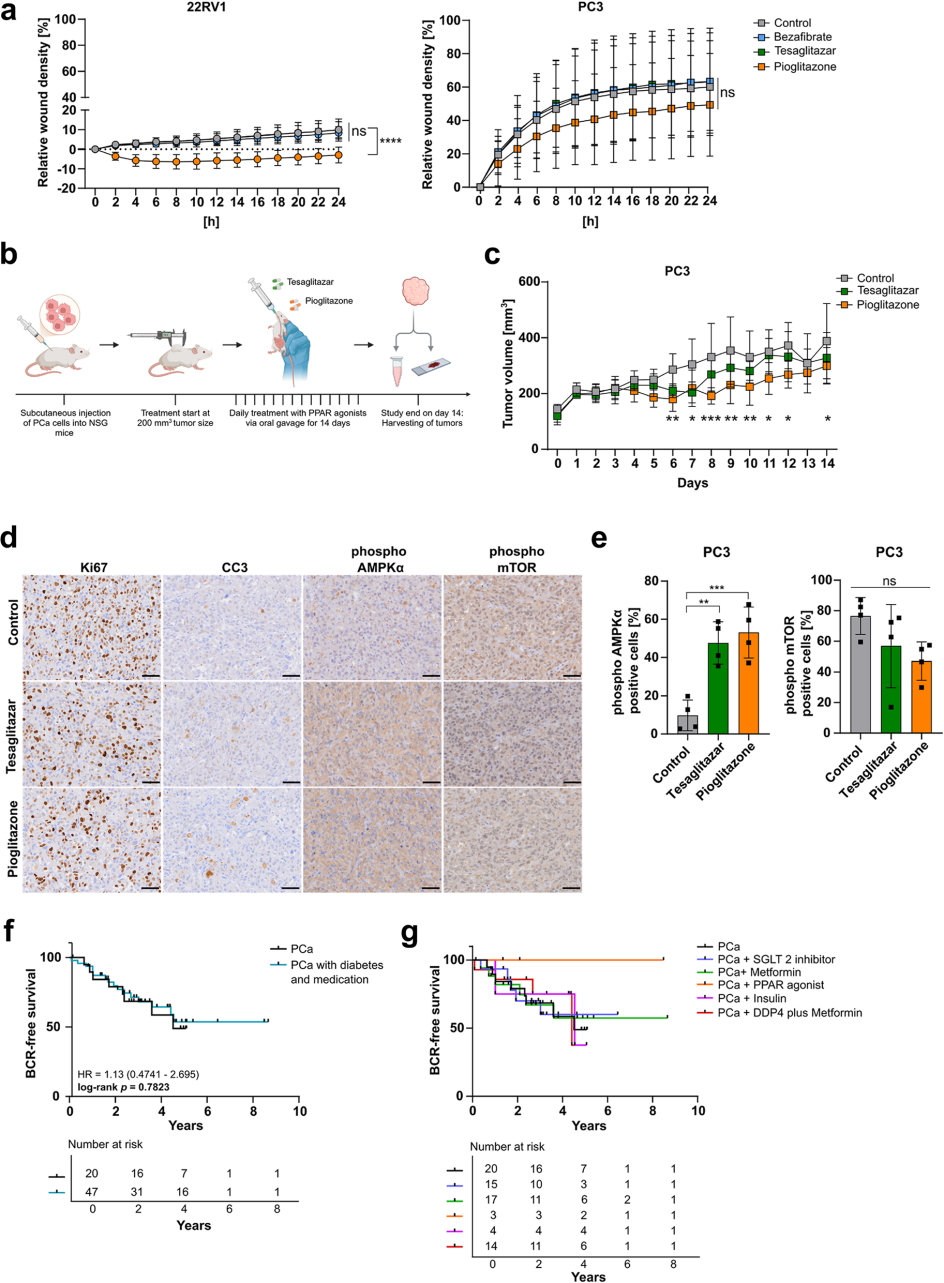
Pioglitazone suppresses cell migration of PCa cells and growth of metastatic PC3 xenograft tumors. **a** Relative wound density resulting from scratch wound assay of 22RV1 (left) and PC3 (right) cells for 24 hours treatment with each PPAR agonist (100 µM, vehicle control = 0.2 % DMSO). One-way ANOVA was used to test for significance (ns = not significant *p* > 0.05, * *p *≤ 0.05, ** *p *≤ 0.01, *** *p *≤ 0.001, **** *p *≤ 0.0001). Data represent the means ± SD of biological triplicates. **b** Workflow scheme of the mouse xenograft experiment of PC3 cells in male NSG mice. **c** Tumor volume throughout the 14 days of treatment with Tesaglitazar (0.4 mg/kg) and Pioglitazone (10 mg/kg) or vehicle control (20 % hydroxypropyl-beta cyclodextrin) (n = 6). Significance was evaluated by two-way ANOVA (ns = not significant *p *> 0.05, * *p *≤ 0.05, ** *p *≤ 0.01, *** *p *≤ 0.001, **** *p *≤ 0.0001). **d** Representative images of immunohistochemistry (IHC) evaluation of Ki67, CC3, phospho AMPKα, and phospho mTOR in xenograft tumors after treatment with Tesaglitazar, Pioglitazone or vehicle control (20x magnification, scale bar = 50 µm). **e** IHC quantifications of phospho AMPKα, and phospho mTOR of Tesaglitazar and Pioglitazone treated xenograft tumors (n = 4). Significance was evaluated by one-way ANOVA (ns = not significant *p *> 0.05, * *p *≤ 0.05, ** *p *≤ 0.01, *** *p *≤ 0.001, **** *p *≤ 0.0001). **f, g** Kaplan-Meier analysis showing BCR-free survival of age-matched non-diabetic (n = 20) and diabetic PCa patients (n = 47) (**f**), as well as diabetic patients treated with SGLT2 inhibitors (n = 15), Metformin (n = 17), PPAR agonists (n = 3), Insulin (n = 4) and DDP4 plus Metformin (n = 14) (**g**). Log-rank (Mantel-Cox) test was used to test for significance (*p *≤ 0.05)

In a scratch wound assay, we assessed the impact of PPAR agonists on the migration of primary and metastatic PCa cells. Pioglitazone significantly reduced relative wound density in the 22RV1 cells. At the same time, a trend (*p* = 0.0689) of reduced cell migration was observed in the metastatic PC3 cells (Fig. 6a, Suppl. Figure 5a). To evaluate the impact of the PPARγ agonists Tesaglitazar and Pioglitazone in vivo*,* we performed a xenograft experiment with the PPARγ-expressing, metastatic PC3 cells. The tumor-bearing NSG mice were treated daily with Tesaglitazar, Pioglitazone and vehicle control via oral gavage for 14 days (Fig. 6b). We discovered that Pioglitazone significantly reduced tumor volume after day 6 of treatment, while Tesaglitazar did not show any significant impact (Fig. 6c, Suppl. Figure 5b). At the endpoint measurement, Pioglitazone-treated mice also showed a trend of reduced tumor weight (*p* = 0.1599) (Suppl. Figure 5c). Furthermore, IHC analysis revealed no difference in the proliferation marker Ki67 and only a slight increase in the apoptosis marker cleaved caspase 3 (CC3) (Fig. 6d, Suppl. Figure 5 d). However, Tesaglitazar and Pioglitazone treatment resulted in a significant increase in phospho AMPKα and a trend of reduced phospho mTOR (*p* = 0.0908) upon Pioglitazone treatment, reflecting the results of the cell culture experiments (Fig. 6d, e). To assess the potential clinical significance of our findings, we finally investigated the relationship between T2D and PCa progression in an age-matched cohort of PCa patients who underwent radical prostatectomy (RP). We compared non-diabetic PCa patients with diabetic PCa patients who used medication for T2D. KM analysis comparing BCR-free survival of these two patient cohorts did not result in any significant difference overall. Nonetheless, it indicated a modest increase in the mean hazard ratio for non-diabetic PCa patients (HR = 1.13) five to ten years after RP (Fig. 6f). Subgroup analysis of diabetic patients receiving various T2D treatments (sodium-glucose cotransporter- 2 (SGLT-2) inhibitors, Metformin, PPAR agonists, insulin, or Dipeptidylpeptidase 4 (DPP4) plus Metformin showed no significant differences in BCR-free survival compared to non-diabetic PCa patients (Fig. 6g, Tab. 2). Surprisingly, diabetic PCa patients treated with PPAR agonists implied no BCR post-RP up to the time of data acquisition. Comparing these patients to non-diabetic PCa patients revealed an increased but insignificant hazard ratio (HR = 3.14) (Tab. 2).

**Table 2:** Comparison of non-diabetic and diabetic PCa patients. The Kaplan–Meier (KM) analysis compared non-diabetic (*n* = 20) with diabetic PCa patients treated with T2D medications (*n *= 53). Significance was assessed via the log-rank (Mantel-Cox, *p* ≤ 0.05) test

Group	Median BCR-free survival	Hazard ratio	95%-CI	*p*-value (log-rank)
PCa (*n* = 20)	4.512	-	-	-
PCa + SGLT2 inhibitor (*n* = 15)	undefined	1.031	0.3361–3.161	0.9578
PCa + Metformin (*n* = 17)	undefined	1.077	0.3742–3.098	0.8911
PCa + PPAR agonist (*n* = 3)	undefined	3.14	0.3601–27.38	0.3003
PCa + Insulin (*n* = 4)	4.539	1.021	0.2160–4.824	0.9792
PCa + DDP4 plus Metformin (*n* = 14)	4.416	1.187	0.3613–3.898	0.7778

## Discussion

Conflicting evidence complicates our understanding of the relation between PCa and T2D in men. Studies indicate that PCa patients with T2D may be protected from PCa disease progression, but mortality rates tend to be higher when T2D is not effectively treated [13–15, 18]. Alongside Metformin and other T2D medication, the PPARγ agonist Pioglitazone is employed either as a monotherapy or combined with Sulfonylureas, Metformin, or insulin for treating T2D [28]. Despite widespread use, a definite link between Pioglitazone and PCa risk remains elusive [52–54]. Our findings demonstrate that *PPARG* is highly expressed in ARPC and associated with worse survival outcomes. These results corroborate earlier reports by Rogenhofer et al., 2012 and Elix et al., 2020, who noted elevated PPARγ levels in PCa tissue compared to benign intraepithelial tissue [55, 56]. We also observed that the PPARγ agonist Pioglitazone significantly reduced cell proliferation in vitro and the growth of metastatic PC3 xenograft tumors. This effect was associated with a reduction in PPARγ protein levels in the PC3 cells and the emergence of an epithelial phenotype in 22RV1 cells. Following our data, Ahmad et al., 2016 reported that PPARγ overexpression in a PTEN-deficient PCa mouse model not only enhanced tumor growth and metastasis formation but was also associated with worse survival for patients while upregulating proteins of lipid metabolism such as FASN [57]. Conversely, the transient knockdown of PPARγ or exposure to PPARγ agonists GW9662, Rosiglitazone, or Pioglitazone reduced PCa tumor growth both in vivo and in vitro while increasing the expression of the epithelial marker E-Cadherin [57–60]. In our study, Pioglitazone significantly altered the proteome landscape of 22RV1 and PC3 cells and induced metabolic shifts dependent on the basal metabolic activity of the cell line. This metabolic reprogramming reduced ATP-synthase-linked mitochondrial ATP production, increased phospho AMPKα and reduced phospho mTOR levels in vivo. Our results recapitulate previous studies in hypoxic HepG2 cells, in which Pioglitazone enhanced ROS production, promoted cell death, and mediated metabolic alterations in lung cancer cells [61–63]. Thereby, squamous lesions were reduced, and an epithelial phenotype in a murine model of squamous lung carcinoma was fostered [61–63]. Furthermore, we discovered that diabetic PCa patients treated with PPAR agonists did not show BCR up to date of the data acquisition. However, due to the small patient size and the lack of statistical significance, this result only implies that there might be a protective effect of PPAR agonists on diabetic PCa patients, which would require further confirmation in a bigger cohort. In addition to PPARγ agonists and Metformin, other diabetic drugs such as DPP4 inhibitors, SGLT-2 inhibitors, or glucagon-like peptide 1 (GLP-1) receptor agonists have been investigated in different cancer types. Several studies have demonstrated that Metformin significantly lowers the incidence of cancer types such as gastric, colorectal, liver, breast, and prostate [64, 65]. This effect was attributed to the disruption of the tricarboxylic acid (TCA) cycle via the liver kinase B1 (LKB1)/AMPKα signaling pathway [17, 64, 66]. Other T2D medications, such as DPP4 inhibitors, enhance insulin secretion after food uptake by preventing GLP-1 degradation, while SGLT-2 inhibitors increase glucose secretion with urine and lower blood glucose levels independent of insulin [65, 67]. Recently, Villani et al., 2016 and Shiba et al., 2018 showed that the SGLT-2 inhibitors Dapagliflozin and Cangliflozin suppress mitochondrial respiration, reduce cell proliferation in lung and PCa cell models and the risk of hepatocellular carcinoma [69, 70]. Despite the beneficial effects of these anti-diabetic drugs on various cancer types, there are still concerns regarding their oncogenic potential. For instance, Pioglitazone has been linked with an elevated risk of bladder cancer, DDP4 inhibitors, and GLP-1 receptor agonists with an increased incidence of pancreatitis, pancreatic, thyroid, ovarian, and PCa, and long-term treatment with high doses of SGLT-2 inhibitors has been associated with renal tumors in rodents [65, 68, 71–74]. Of note, in our study, as in several others, micromolar concentrations of the PPAR agonists have been used [75]. Even though these concentrations were not toxic, it has been shown that PPAR agonists can also activate off-target pathways, such as AMPKα, PI3K/AKT, NF-κB, or VEGF [75]. This might be caused by the activation of other nuclear receptors, such as retinoid-X-receptor or the response to the drug-induced metabolic stress independent of PPAR expression [75]. These observations raise critical questions about whether targeting metabolic pathways can universally benefit cancer patients or if these effects are cancer-specific. It is known that PCa undergoes metabolic reprogramming from healthy to primary and metastatic stages due to the altered zinc levels in the peripheral zone of the prostate [76, 77]. High zinc levels inhibit the mitochondrial aconitase in healthy prostate cells, thereby truncating the TCA cycle and promoting aerobic glycolysis, known as the Warburg effect [78–81]. In primary PCa, zinc levels decrease due to the loss of zinc receptors, which reactivates aconitase and the TCA cycle, while glycolysis is suppressed. In CRPCa and post-ADT, a second metabolic shift occurs, which elevates aerobic glycolysis [78–81]. These differences in the basal metabolome of primary and metastatic PCa, were also reflected in the cell models of our study. Besides these metabolic shifts during PCa progression, it has also been shown that AR signaling promotes both lipogenesis and OXPHOS, leading to increased cell proliferation in primary PCa [76, 82]. Targeting these metabolic vulnerabilities in PCa offers promising therapeutic options for novel PCa therapies. Specifically, inhibitors of de novo lipogenesis and FASN (TVB-3166, TVB-2640) suppressed tumor growth in xenografts of 22RV1 cells, and in combination therapy with Paclitaxel, the effect was amplified by 97% [83–85]. The FASN inhibitor TVB-2640, alongside the acetyl-CoA carboxylase inhibitors Firscostat and PF-05175157, originally developed for non-alcoholic fatty liver disease, is currently undergoing clinical trials for PCa therapy [83–85]. Moreover, combined therapies of Enzalutamide with the FAO inhibitors Ranolazine and Perhexilin, primarily used for angina pectoris, reduced tumor growth in vitro and in vivo [86]. Additionally, clinical trials of the glutaminolysis inhibitor CB-839 in combination with the poly-ADP ribose polymerase inhibitor Talazoparib are exploring therapeutic strategies for metastatic CRPCa patients [87].

In conclusion, our study uncovers that the anti-diabetic PPARγ agonist Pioglitazone reduced cell proliferation in vitro and in vivo by inducing metabolic reprogramming in primary and metastatic PCa cells. This resulted in a decreased ATP synthase-coupled mitochondrial ATP production and cell migration, while additionally in primary PCa an epithelial phenotype is induced. Our findings position Pioglitazone and similar metabolic inhibitors at the forefront of emerging therapeutic strategies for PCa. However, further in-depth and long-term longitudinal studies will be essential to fully elucidate the impact of these metabolic inhibitors on the development and progression of PCa and patient survival.

## Supplementary Information

Supplementary Material 1. Supplementary figures and legends.

Supplementary Material 2. Table S1 Excel file containing proteomics data comparing basal differences between 22RV1 and PC3 cells.

Supplementary Material 3. Table S2 Excel file containing enrichment analysis of basal 22RV1 versus PC3.

Supplementary Material 4. Table S3 Excel file containing GSEA data comparing basal and PPAR agonist-induced effects.

Supplementary Material 5. Table S4 Excel file containing proteomics data of PPAR agonists treated 22RV1.

Supplementary Material 6. Table S5 Excel file containing proteomics data of PPAR agonists treated PC3.

Supplementary Material 7. Table S6 Excel file containing NMR metabolomics data of PPAR treated 22RV1 and PC3.

## Data Availability

The following publicly available datasets were used: RNA-Seq data from the Prostate Cancer Atlas (https://prostatecanceratlas.org/) and The Cancer Genome Atlas-Prostate Adenocarcinoma (https://portal.gdc.cancer.gov/projects/TCGA-PRAD). Proteomics and metabolomics analysis data are available as supplementary material for this publication. All raw proteomics data were deposited to the ProteomeXchange Consortium via the PRIDE partner repository with the dataset identifier PXD060526.

## Abbreviations

ACN: Acetonitrile
Adj. *p*-value: Adjusted *p*-value
ADT: Androgen deprivation therapy
AR: Androgen receptor
AR-FL: Androgen receptor full-length
ARPC: Androgen receptor pathway independent prostate cancer
AR-V7: Androgen receptor splice variant 7
BCR: Biochemical recurrence
CC3: Cleaved caspase 3
CREB: CAMP responsive element binding protein
CRPCa: Castration-resistant prostate cancer
DDP4: Dipeptidylpeptidase 4
DEP: Differentially expressed proteins
DHT: 5α-Dihydrotestosterone
DLD: Dihydrolipoamide dehydrogenase
ECAR: Extracellular acidification rate
EMT: Epithelial to mesenchymal transition
ETC: Electron transport chain
FA: Formic acid
FAO: Fatty acid oxidation
FASN: Fatty acid synthase
FCS: Fetal calf serum
FDG: Fluoro- 2-deoxy-D-glucose
FFPE: Formalin-fixed and paraffin-embedded
FTHA: Fluoro- 6-thia-heptadecanoic acid
G6PD: Glucose- 6-phosphate dehydrogenase
GLP1: Glucagon-like peptide 1
GSEA: Gene set enrichment analysis
GSR: Glutathione-disulfide reductase
HbA1c: Hemoglobin A1c
HK1: Hexokinase 1
IF: Immunofluorescence
IHC: Immunohistochemistry
KM: Kaplan-Meier
LKB1: Liver kinase B1
ME2: Malic enzyme 2
mTORC1: Mechanistic target of rapamycin kinase complex 1
nLC-MS/MS: Nano-liquid chromatography-mass spectrometry
NMR: Nuclear magnetic resonance
OCR: Oxygen consumption rate
OXPHOS: Oxidative phosphorylation
PARP: Poly ADP ribose polymerase
PCa: Prostate cancer
Pen/Strep: Penicillin/Streptomycin
PIN: Prostatic intraepithelial neoplasia
PLS-DA: Partial least squares-discriminant analysis
PPARα: Peroxisome proliferator-activated receptor alpha
PPARγ: Peroxisome proliferator-activated receptor gamma
PPARδ: Peroxisome proliferator-activated receptor delta
PTEN: Phosphatase and tensin homolog
qRT-PCR: Quantitative reverse transcription polymerase chain reaction
RT: Room temperature
ROS: Reactive oxygen species
SD: Standard deviation
SGLT2: Sodium-glucose cotransporter- 2
STAT3: Signal transducer and activator of transcription 3
T2D: Type 2 diabetes
TCA: Tricarboxylic acid
TGF-beta: Transforming growth factor-beta
TZD: Thiazolidinediones
VIM: Vimentin
