# Supplementary material for: The anti-diabetic PPARγ agonist Pioglitazone inhibits cell proliferation and induces metabolic reprogramming in prostate cancer
Source: Mol Cancer. 2025 May 5;24:134. doi: 10.1186/s12943-025-02320-y (PMC12051277; doi:10.1186/s12943-025-02320-y)
Supplement: Supplementary file 1 — Supplementary Material 1. Supplementary figures and legends. [file 12943_2025_2320_MOESM1_ESM.docx]

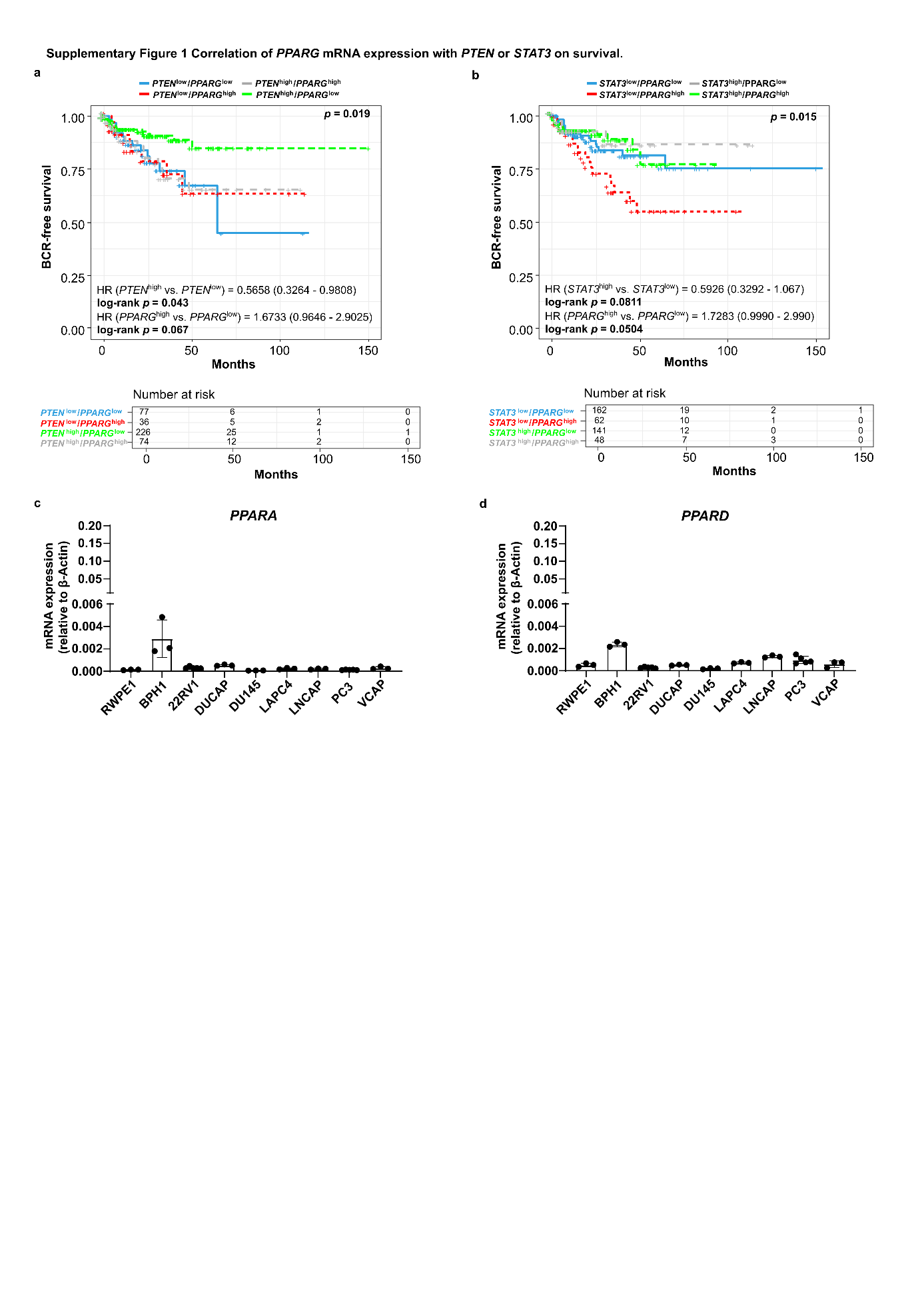

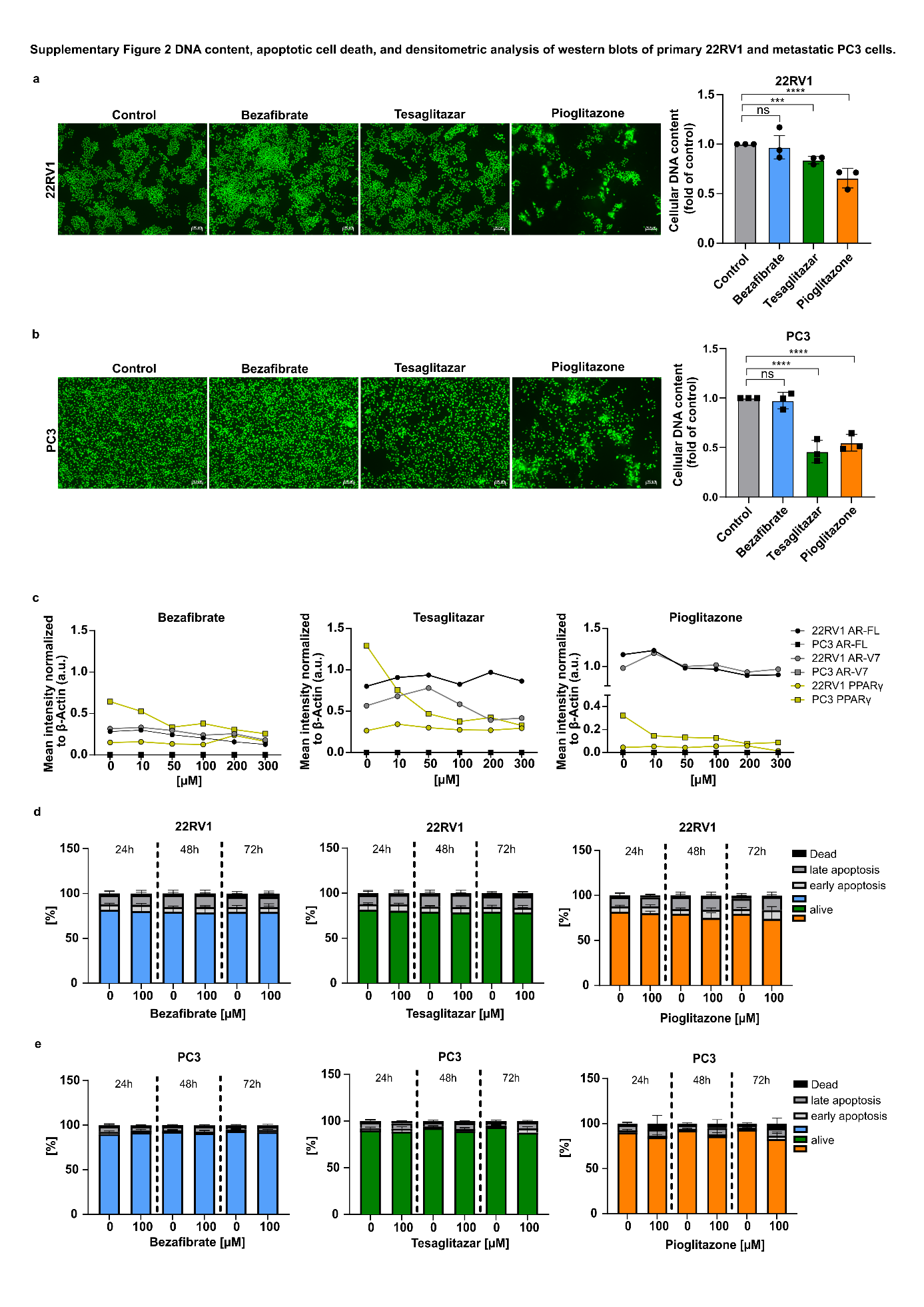

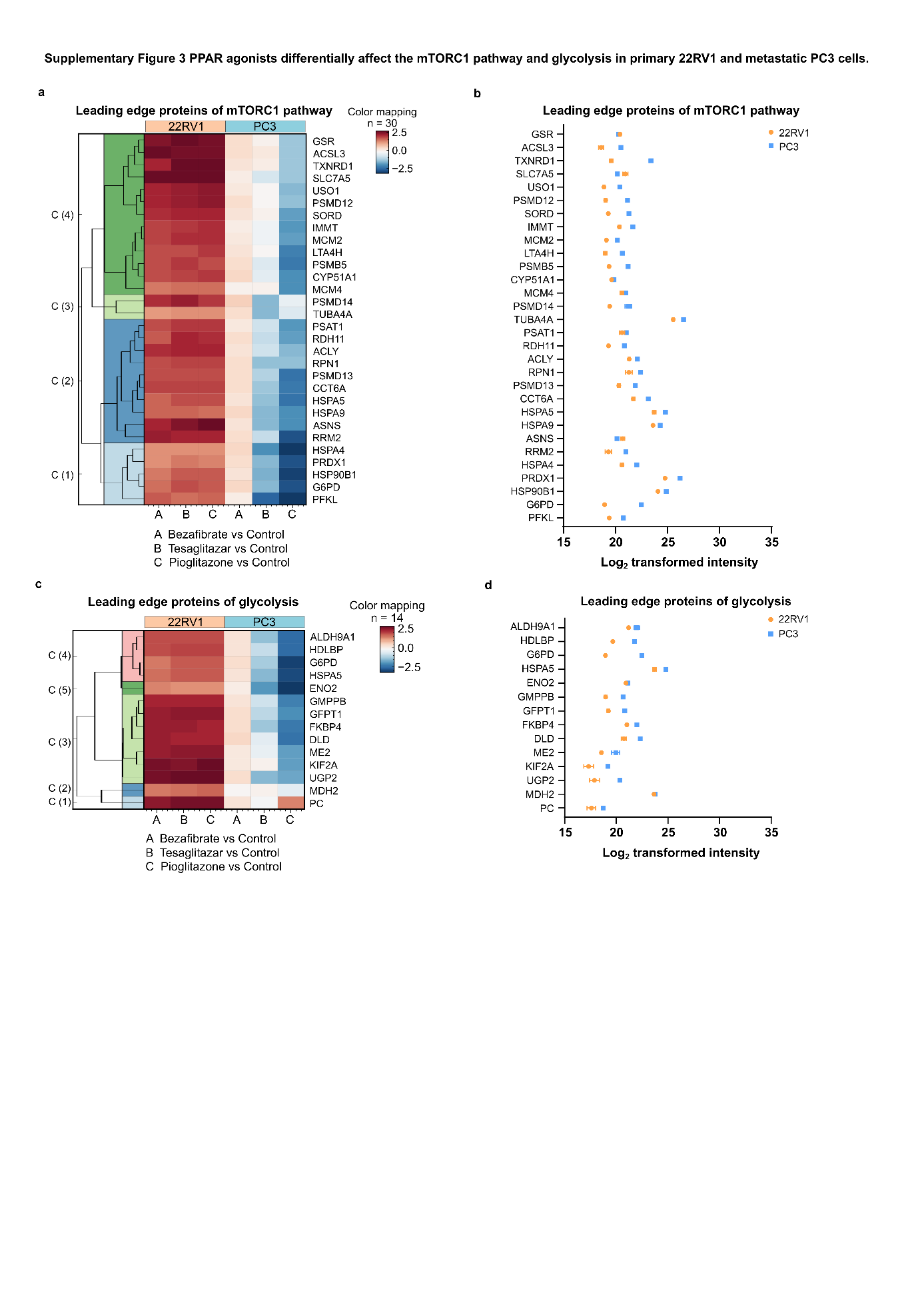

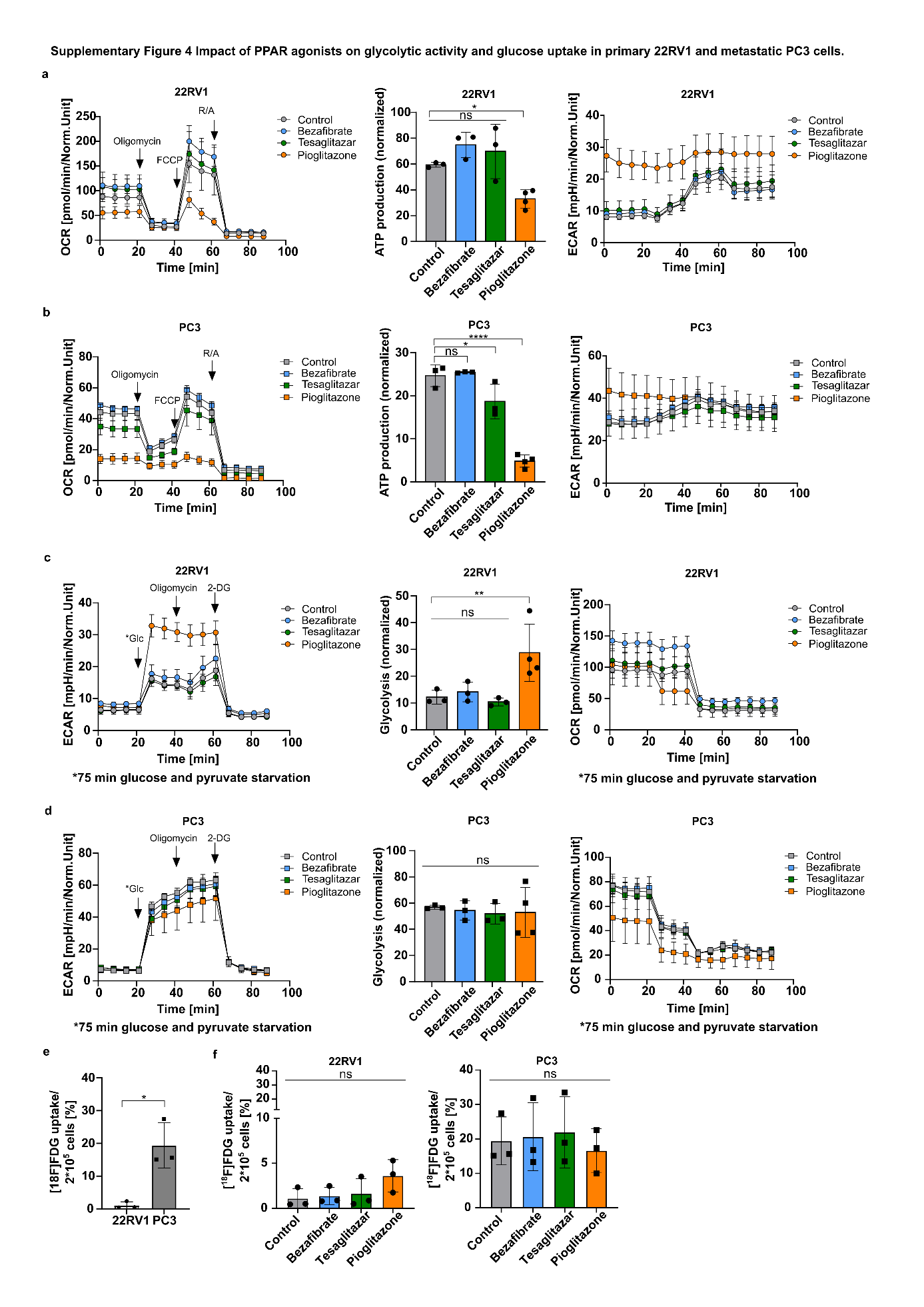

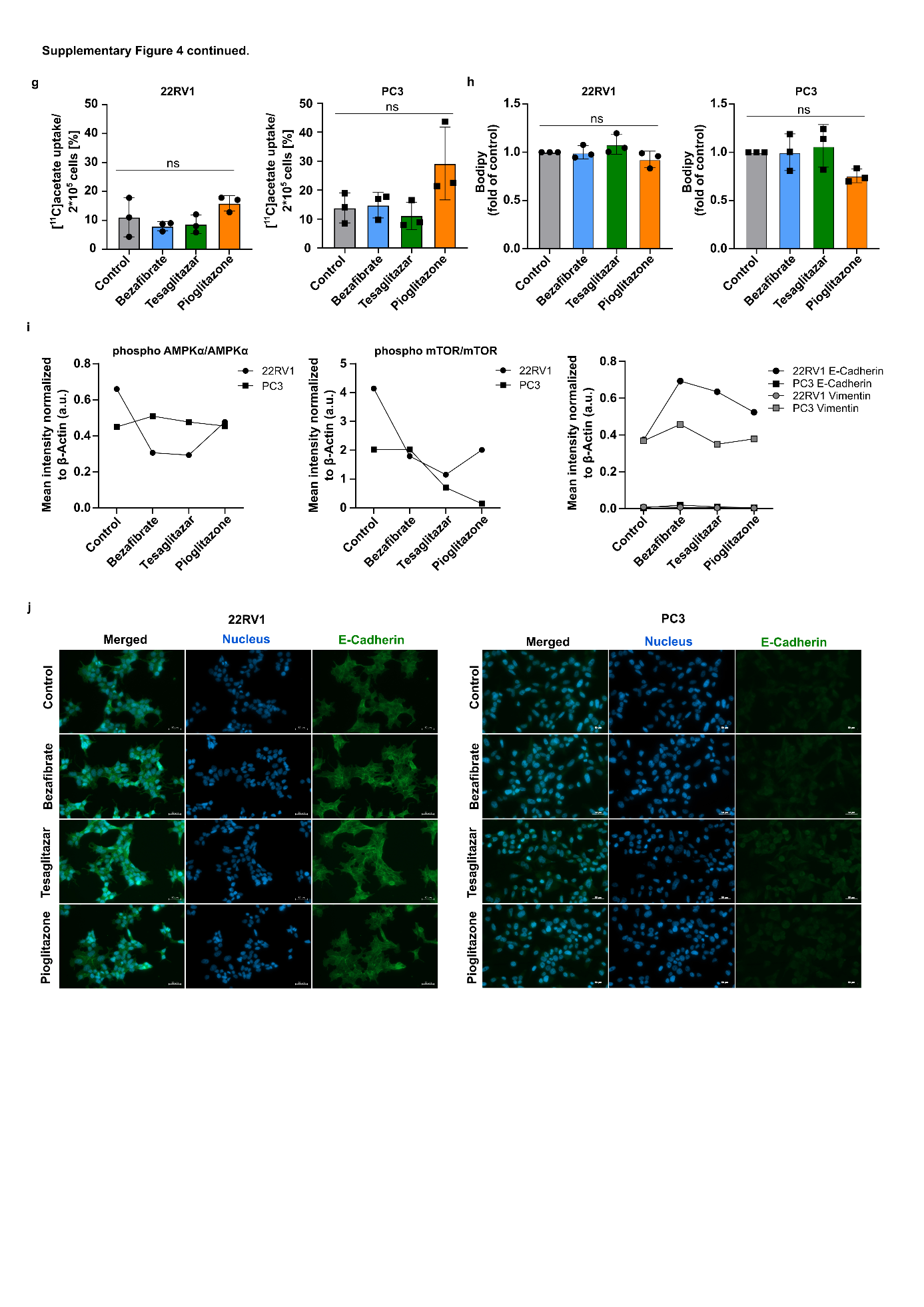

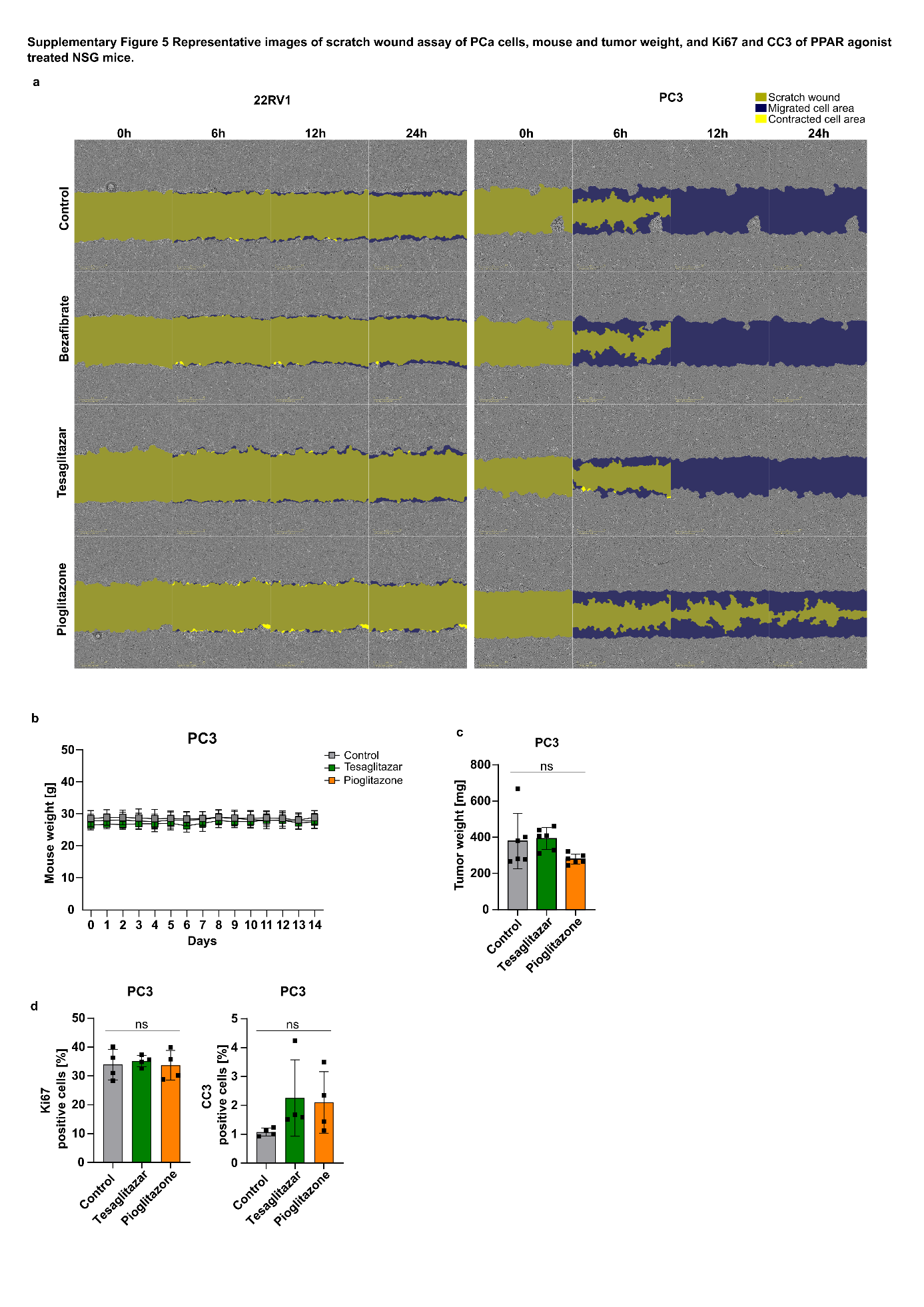


**Supplementary Figure 1 Correlation of *PPARG* expression with *PTEN* or *STAT3* on survival.** **a, b** Kaplan-Meier analysis showing the effect of *PPARG* expression on BCR-free survival of PCa patients from the TCGA-PRAD cohort (n = 333) upon loss of *PTEN* (**a**) or *STAT3* (**b**). Cox regression was used to test for significance (*p*≤0.05). **c, d** QRT-PCR analysis displaying basal mRNA expression of *PPARA* (**c**) and *PPARD* (**d**) relative to the β-Actin in different PCa cell lines. Data are representative of the means ± SD of biological triplicates.

**Supplementary Figure 2 DNA content, apoptotic cell death, and densitometric analysis of western blots of primary 22RV1 and metastatic PC3 cells. a, b** DNA content and respective immunofluorescence images (green, FITC channel, 10x magnification, scale bar = 100 µm) of 22RV1 (**a**) and PC3 (**b**) cells following treatment with PPAR agonists Bezafibrate, Tesaglitazar, and Pioglitazone (72 hours, 100 µM) or vehicle control (0.2 % DMSO). **c** Densitometric analysis of western blots of main figure 2 displaying quantifications of AR-FL, AR-V7, and PPARγ normalized to β-Actin (a.u. = arbitrary units). **d, e** Flow cytometry-based apoptotic cell death analysis of 22RV1 (**d**) and PC3 (**e**) cells treated with PPAR agonists Bezafibrate (left), Tesaglitazar (middle), and Pioglitazone (right) (100 µM) for 24 to 72 hours assessed by Annexin V only (early apoptotic) or Annexin V and DAPI (late apoptotic) staining. One-way ANOVA was used to test for significance (ns = not significant *p* > 0.05, * *p* ≤ 0.05, ** *p* ≤ 0.01, *** *p* ≤ 0.001, **** *p* ≤ 0.0001). Data are representative of the means ± SD of biological triplicates.

**Supplementary Figure 3** **PPAR agonists differentially affect the mTORC1 pathway and glycolysis in primary 22RV1 and metastatic PC3 cells.** **a, c** Heatmap summarizing leading-edge proteins resulting from the GSEA analysis of mTORC1 pathway (**a**) and glycolysis (**c**) with log_2_ fold changes comparing each PPAR agonist with control Pioglitazone (24 hours, 100 µM, vehicle control = 0.2 % DMSO) **b, d** Mean basal protein abundance levels (log_2_ transformed intensities) of leading-edge proteins assigned to mTORC1 pathway (**b**) and glycolysis (**d**) in 22RV1 (orange) and PC3 (blue) cells.

**Supplementary Figure 4 Impact of PPAR agonists on glycolytic activity and glucose uptake in primary 22RV1 and metastatic PC3 cells. a, b** Mitochondrial stress-induced normalized OCR, ECAR responses, and ATP production in 22RV1 (**a**) and PC3 (**b**) cells under control conditions and following PPAR agonist Bezafibrate, Tesaglitazar, and Pioglitazone (24 hours, 50 µM, vehicle control = 0.2 % DMSO) treatment. **c, d** Glycolysis test after 75 minutes glucose and pyruvate starvation showing normalized ECAR, OCR, and glycolysis of 22RV1 (**c**) and PC3 (**d**) cells treated with PPAR agonists. **e** Basal [^18^F]FDG uptake of 22RV1 and PC3 cells. **f** [^18^F]FDG uptake after 24 hours of treatment with each PPAR agonist in 22RV1 and PC3 cells. **g** [^11^C]acetate uptake after 24 hours of treatment with PPAR agonist Bezafibrate, Tesaglitazar, and Pioglitazone (100 µM, vehicle control = 0.2 % DMSO) in 22RV1 and PC3 cells. **h** Flow cytometry analysis of Bodipy 493/503 stained 22RV1 and PC3 cells upon treatment with each PPAR agonist. **i** Densitometric analysis of western blots of main figure 5 displaying quantifications of E-Cadherin, vimentin, the ratio of phospho AMPKα/AMPKα, and phospho mTOR/mTOR normalized to β-Actin (a.u. = arbitrary units). **j** Representative immunofluorescence images of E-Cadherin (green, 40x magnification) and DAPI stained nuclei (blue, 40x magnification, scale bar = 50 µm) of 22RV1 (left) and PC3 cells (right) upon treatment with each PPAR agonists. One-way ANOVA was used to test for significance (ns = not significant *p* > 0.05, * *p* ≤ 0.05, ** *p* ≤ 0.01, *** *p* ≤ 0.001, **** *p* ≤ 0.0001). Data are representative of the means ± SD of biological triplicates. One representative experiment of the Seahorse assay is shown.

**Supplementary Figure 5 Representative images of scratch wound assay of PCa cells and weight of PPAR agonist treated NSG mice. a** Brightfield images (10x magnification, top, scale bar = 400 µm) from scratch wound assay in 22RV1 (left) and PC3 (right) during treatment with each PPAR agonist (24 hours, 100 µM, vehicle control = 0.2 % DMSO). **b** Weight of NSG mice injected with PC3 cells throughout the 14 days of treatment with Tesaglitazar (0.4 mg/kg) and Pioglitazone (10 mg/kg) or vehicle control (20 % hydroxypropyl-beta cyclodextrin) (n = 6). **c** Tumor weight measured at endpoint (day 14) of vehicle, Tesaglitazar, and Pioglitazone treated NSG mice (n = 6). **d** IHC quantifications of Ki67 and CC3 of Tesaglitazar and Pioglitazone treated xenograft tumors (n = 4). Significance was evaluated by one-way ANOVA (ns = not significant *p* > 0.05, * *p* ≤ 0.05, ** *p* ≤ 0.01, *** *p* ≤ 0.001, **** *p* ≤ 0.0001).
